# *TP53* Mutations Promote Immunogenic Activity in Breast Cancer

**DOI:** 10.1155/2019/5952836

**Published:** 2019-06-02

**Authors:** Zhixian Liu, Zehang Jiang, Yingsheng Gao, Lirui Wang, Cai Chen, Xiaosheng Wang

**Affiliations:** ^1^Biomedical Informatics Research Lab, School of Basic Medicine and Clinical Pharmacy, China Pharmaceutical University, Nanjing 211198, China; ^2^Cancer Genomics Research Center, School of Basic Medicine and Clinical Pharmacy, China Pharmaceutical University, Nanjing 211198, China; ^3^Big Data Research Institute, China Pharmaceutical University, Nanjing 211198, China; ^4^Department of Basic Medicine, School of Basic Medicine and Clinical Pharmacy, China Pharmaceutical University, Nanjing 211198, China; ^5^Department of Electrical and Computer Engineering, University of California, San Diego, La Jolla, CA 92093, USA

## Abstract

**Background:**

Although immunotherapy has recently achieved clinical successes in a variety of cancers, thus far there is no immunotherapeutic strategy for breast cancer (BC). Thus, it is important to discover biomarkers for identifying BC patients responsive to immunotherapy.* TP53* mutations were often associated with worse clinical outcomes in BC whose triple-negative subtype has a high* TP53* mutation rate (approximately 80%). To explore a potentially promising therapeutic option for the* TP53*-mutated BC subtype, we studied the association between* TP53* mutations and immunogenic activity in BC.

**Methods:**

We compared the enrichment levels of 26 immune signatures that indicated activities of diverse immune cells, functions, and pathways between* TP53*-mutated and* TP53*-wildtype BCs based on two large-scale BC multiomics datasets. Moreover, we explored the molecular cues associated with the differences in immunogenic activity between* TP53*-mutated and* TP53*-wildtype BCs. Furthermore, we performed experimental validation of the findings from bioinformatics analysis.

**Results:**

Bioinformatics analysis showed that almost all analyzed immune signatures showed significantly higher enrichment levels in* TP53*-mutated BCs than in* TP53*-wildtype BCs. Moreover,* in vitro* experiments confirmed that mutant p53 could increase BC immunogenicity. Both computational and experimental results demonstrated that* TP53* mutations could promote BC immunogenicity* via* regulation of the p53-mediated pathways including cell cycle, apoptosis, Wnt, Jak-STAT, NOD-like receptor, and glycolysis. Furthermore, we found that elevated immune activity was likely associated with a better survival prognosis in* TP53*-mutated BCs, but not necessarily in* TP53*-wildtype BCs.

**Conclusions:**

* TP53* mutations may promote immunogenic activity in BC, suggesting that the* TP53* mutation status could be a useful biomarker for stratifying BC patients responsive to immunotherapy.

## 1. Introduction

The tumor suppressor p53 plays an important role in the regulation of cell-cycle, apoptosis, DNA repair, cellular senescence, and autophagy [1]. Accordingly,* TP53 *mutations and dysfunction are importantly involved in carcinogenesis due to the disturbance of these biologic processes it functions in. In fact,* TP53 *mutations occur in more than half of all human cancer cases [2] and are independent markers of poor prognosis in a variety of cancers [3]. p53 also plays an important role in immune regulation, e.g., the control of immune responses to infection, autoimmunity, and cancer [4]. p53 functions in immunity by induction of apoptosis, removal of apoptotic cells, antiviral defense, induction of type I IFN, enhanced pathogen recognition, cytokine production, and immune checkpoint regulation [4]. Several studies have explored the association of p53 and tumor immune regulation [5–8]. For example, the p53 activation in the tumor microenvironment (TME) might overcome tumor immune suppression and enhance antitumor immunity [8]. p53 could transactivate many tumor immunosuppressive genes such as* PD-L1*,* VISTA*, and* FOXP3* [5]. p53 functioned in both tumor suppression and anticancer immunosurveillance via regulation of* VISTA* [5].

Recently, cancer immunotherapy has shown successes in treating various cancers [9]. In particular, the blockade of immune checkpoints has achieved rapid clinical successes in multiple cancers, including skin, lung, kidney, bladder, head and neck cancers, lymphoma, and the cancers with deficient DNA mismatch repair (dMMR) [10]. Unfortunately, current immunotherapies are only propitious to a subset of cancer patients [11]. Some molecular biomarkers associated with cancer immunotherapy response have been identified, e.g., tumor mutation burden (TMB) [12], neoantigens [13], dMMR [14], and PD-L1 expression [15]. However, few studies have correlated the* TP53* mutation status with cancer immunotherapy response, although a recent clinical trial (phase II) data showed that patients with mutated-p53 metastatic breast cancer had better overall survival (OS) when treated with the immunooncology viral agent REOLYSIN® in combination with paclitaxel [16].

Breast cancer (BC) is the most common cancer and the second leading cause of cancer death in women [17]. The triple-negative BC (TNBC) is the BC subtype which does not express estrogen receptor (ER) and progesterone receptor (PR) and lacks overexpression of the human epidermal growth factor receptor 2 (HER2) [18]. TNBC has a high* TP53* mutation rate (80% in TNBC versus 33% in general BC) [19] and has a poor prognosis due to its aggressive clinical behavior and lack of response to hormonal or HER2 receptor-targeted therapy. Although there is currently no immunotherapeutic drug clinically used for BC therapy, several studies have indicated that TNBC might be propitious to immunotherapy [20–22].

To explore the association of* TP53* mutations with tumor immunity in BC, we compared the activity of 26 immune signatures between* TP53*-mutated and* TP53*-wildtype BCs based on the Cancer Genome Atlas (TCGA) [23] and METABRIC [24] BC genomic data. We found that these immune signatures exhibited significantly higher activity in* TP53*-mutated BCs than in* TP53*-wildtype BCs. Furthermore, we explored the molecular cues correlated with the differences in immune activities between* TP53*-mutated and* TP53*-wildtype BCs. Finally, we performed experimental validation of the findings from bioinformatics analysis.

## 2. Methods

### 2.1. Datasets

We downloaded TCGA BC RNA-Seq gene expression profiles (Level 3), gene somatic mutations (Level 3), somatic copy number alterations (SCNAs) (Level 3), protein expression profiles (Level 3), and clinical data from the genomic data commons data portal (https://portal.gdc.cancer.gov/), and the METABRIC gene expression profiles, gene somatic mutations, SCNAs, and clinical data from cBioPortal (http://www.cbioportal.org). We obtained 26 gene sets representing 26 different immune signatures from several publications, including 15 immune cell types and functions [25], tumor-infiltrating lymphocytes (TILs) [26], proinflammatory [27], parainflammation (PI) [28], cytokine and cytokine receptor (CCR) [29], human leukocyte antigen (HLA) [22], cancer testis (CT) antigen [30], regulatory T (Treg) cells [31], immune checkpoint [31], metastasis-promoting, and metastasis-inhibiting [32] (Supplementary Table S1). The sample sizes of breast cancers are presented in Supplementary Table S2. We performed computational and statistical analyses using R programming (https://www.r-project.org/).

### 2.2. Comparisons of Gene Expression Levels, Gene-Set Enrichment Levels, and Protein Expression Levels between Two Classes of Samples

We normalized the TCGA BC gene expression values by base-2 log transformation and used the downloaded normalized METABRIC gene expression data. For the TCGA BC protein expression profiles data, we used the downloaded normalized data. We quantified the activity of an immune signature in a sample by the single-sample gene-set enrichment analysis (ssGSEA) (“GSVA” version 1.24.2, R package) score [33, 34] of the gene set representing the immune signature (a higher ssGSEA score indicated a higher activity) (Supplementary Table S3). We compared gene or protein expression levels between two classes of samples using Student's* t *test and compared immune signature enrichment levels (ssGSEA scores) between two classes of samples using the Mann-Whitney U test. The false discovery rate (FDR) was utilized to adjust for multiple tests by the Benjamini and Hochberg (BH) method [35]. The threshold of FDR < 0.05 was used to identify the statistical significance. The comparisons involving normal tissue were performed only in TCGA since METABRIC had no normal tissue related data available.

### 2.3. Comparison of the Immune Cell Infiltration Degree between* TP53*-Mutated and* TP53*-Wildtype BCs

We evaluated the immune cell infiltration degree in BC using ESTIMATE (“estimate” version 1.0.13, R package) [36]. For each BC sample, ESTIMATE output an immune score that quantified its immune cell infiltration degree based on the BC gene expression data. In addition, we obtained the lymphocyte infiltration percentage data for BC from the TCGA BC clinical data. We compared immune scores or lymphocyte infiltration percentage between* TP53*-mutated and* TP53*-wildtype BCs using the Mann-Whitney U test.

### 2.4. Gene-Set Enrichment Analysis

We used GSEA [37] to identify differentially expressed KEGG pathways between* TP53*-mutated and* TP53*-widtype BCs with the threshold of FDR < 0.05.

### 2.5. Comparison of the Proportions of Leukocyte Cell Subsets between* TP53*-Mutated and* TP53*-Wildtype BCs

We used CIBERSORT [38] to calculate the proportions of 22 human leukocyte cell subsets and compared the proportions of the leukocyte cell subsets between* TP53*-mutated and* TP53*-wildtype BCs using the Mann-Whitney U test.

### 2.6. Correlation of Pathway or Protein Activity with Immune Activity in BCs

We obtained the gene-set collections for p53-mediated pathways from KEGG [39] and quantified the pathway activity with the ssGSEA score of the set of genes included in the pathway. To correct for the strong correlation between the p53 pathway and the other p53-mediated pathways, we evaluated the correlation between a pathway activity and an immune activity using the first-order partial correlation method (“ppcor” version 1.1, R package) [40] with the significance level of FDR*<*0.05. The correlation between a protein and an immune signature was evaluated by the Spearman correlation coefficient (“rho”) of the protein expression levels and the immune signature enrichment levels.

### 2.7. Survival Analyses

We compared overall survival (OS) and disease-free survival (DFS) between two classes of BC patients divided by the* TP53 *mutation status (*TP53*-mutated versus* TP53*-wildtype), or the median values of gene expression levels, immune signature enrichment levels, and immune scores. Kaplan-Meier survival curves were used to exhibit the 20-year survival differences between both groups, and the log-rank test was used to evaluate the significance of survival-time differences with a threshold of P < 0.05.

### 2.8. Comparison of Mutation Counts between* TP53*-Mutated and* TP53*-Wildtype BCs

We compared mutation counts (defined as total number of somatic point mutations and indels) between* TP53*-mutated and* TP53*-wildtype BCs using the Mann-Whitney U test. This comparison was performed only in TCGA since somatic mutation data in TCGA were generated by whole exome sequencing while in METABRIC they were generated by targeted exome sequencing.

### 2.9. In Vitro Experiments

#### 2.9.1. Cell Lines and Cell Culture

Human cells from breast cancer, MCF-7 (ER+/HER2-), and natural killer cells NK-92 were from the American Type Culture Collection (ATCC). MCF-7 was cultured in RPMI-1640 (GIBCO, USA) supplemented with 10% fetal bovine serum (FBS, GIBCO, USA). NK92 cells were incubated in *α*-MEM (GIBCO, USA) with 2 mM L-glutamine, 0.2 mM inositol, 0.02 mM folic acid, 0.01 mM 2-mercaptoethanol, 10 ng/ml IL-2, 12.5% FBS, and 12.5% horse serum (GIBCO, USA). These cells were cultured in a humidified incubator at 37°C and a 5% CO_2_ atmosphere and were harvested in logarithmic growth phase.

#### 2.9.2. Cell Transfection

The MCF-7 cells without antibiotic were maintained in the medium with* TP53*-mutated (c.596 G > T, c.818 A > G, and c.925 T > C) virus stock solution and polybrene (5 ug/mL) for 24h. The transfected MCF-7 cells were cultured in a humidified incubator at 37°C and a 5% CO_2_ atmosphere for 48h.

#### 2.9.3. Coculture of MCF-7 and NK-92 Cells

The transwell chamber (Corning Inc., Corning, NY, USA) was inserted into a 6-well plate to construct a coculture system. MCF-7 cells were seeded on the 6-well plate at a density of 5×10^4^ cells/well, and NK-92 cells were seeded on the membrane (polyethylene terephthalate, pore size, 0.4*μ*m) of the transwell chamber at a density of 5×10^4^ cells/chamber. NK-92 and MCF-7 cells were cocultured in a humidified incubator at 37°C and a 5% CO_2_ atmosphere for 24h.

#### 2.9.4. Transwell Migration Assay

After coculture of 24h, NK-92 cells were harvested and resuspended in the upper transwell chambers (8-*μ*m pores, Corning), and MCF-7 cells in the lower 24-well plates. Both NK-92 and MCF-7 cells were incubated at 37°C for 24h. The membrane was removed and its upper surface was wiped away with a cotton swab to remove the unmigrated NK-92 cells. The membrane was fixed in neutral formalin and air-dried at a room temperature and was stained with 0.1% crystal violet at 37°C for 30 min. The number of NK-92 cells that migrated to the lower surface of the membrane was counted under light microscope. Each assay was performed in triplicate wells.

#### 2.9.5. EdU Proliferation Assay

After coculture of 24h, an EdU (5-ethynyl-2′-deoxyuridine, Invitrogen, CA, USA) proliferation assay [41] was performed to measure the proliferation ability of NK-92 cells. NK-92 cells were plated in 96-well plates at a density of 2 × 10^3^ cells/well for 24h. The cells were incubated with 10 *μ*M EdU for 24h at 37°C before fixation, permeabilization, and EdU staining according to manufacturer's protocol. The cell nuclei were stained with DAPI (Sigma) at a concentration of 1 *μ*g/ml for 20 min. The proportion of the cells incorporated EdU was determined with fluorescence microscopy. Each assay was performed in triplicate wells.

#### 2.9.6. CCL4 and CCL5 Enzyme-Linked Immunosorbent Assay

After coculture of 24h, supernatants from NK-92 cells were collected and assayed. CCL4 and CCL5 protein levels were evaluated by ELISA, according to manufacturer's protocol (Shanghai Enzyme-Linked Biotechnology Co., Ltd. China). Study samples and standard dilutions of the chemokines/cytokines were assayed in triplicate. The absorbance was read at 450 nm with the correction set to 570 nm by a microplate reader (BioTek, USA).

#### 2.9.7. Reverse Transcription Quantitative PCR (qPCR) Analysis

Z-DEVD-FMK, abemaciclib, and MPP were purchased from Selleck, Haoyuan Chemexpress, and Cayman, respectively. MCF-7 cells were harvested after being treated by drugs (Z-DEVD-FMK, 50 *μ*M, 72h; abemaciclib, 250 nM, 7 days; MPP, 0.1 nM, 72h). The total RNA was isolated by Trizol (Invitrogen, USA) and was reversely transcribed into cDNA using the RevertAid First Strand cDNA Synthesis Kit (Thermo Fisher, USA). Primer sequences used for qPCR were presented in the Supplementary Table S4. Primers were diluted in nuclease-free water with the Real time PCR Master Mix (SYBR Green) (TOYOBO Co., LTD, JAPAN). Relative copy number was determined by calculating the fold-change difference in the gene of interest relative to *β*-actin. The qPCR was performed on an ABI 7500 FAST and Applied Biosystems StepOnePlus Real Time PCR machine.

#### 2.9.8. Western Blotting

MCF-7 cells were washed twice with cold PBS and were lysed in SDS buffer (1% SDS, 0.1 M Tris pH 7.4, 10% glycerol) supplemented with protease inhibitors. The protein concentration was determined by Bradford Protein Assay (Bio-Rad). After normalization of the total protein content, samples were resolved by standard SDS-PAGE. After Western blotting transfer, NC membranes (Millipore) were incubated with antibodies ER-alpha (21244-1-AP, Proteintech Group, INC.) and cleaved-caspase 3 (KGYC0004-6, KeyGEN Biotech, China). After 2h incubation with the HRP-labeled secondary antibody (KGAA002-1, KeyGEN Biotech, China), proteins were visualized by enhanced chemiluminescence using a G: BOX chemiXR5 digital imaging system.

#### 2.9.9. Knockdown of p53 with Small Interfering RNA (siRNA)

MCF-7 cells were seeded in 6-well plates and grown until they attained 70% confluency. Transfection of siRNA was performed using Lipofectamine 3000 (Invitrogen, CA) according to the manufacturer's instructions. p53 siRNA and control siRNA were synthesized by KeyGEN BioTECH. The sequence of siRNA against p53 was sense (5′-3′): CCAUCCACUACAACUACAUdTdT, and antisense (5′-3′): AUGUAGUUGUAGUGGAUGGdTdG.

#### 2.9.10. Statistical Analyses

All experimental data were expressed as mean ± SD and were analyzed by* t* test using Prism 5.0 software (GraphPad). P < 0.05 was considered statistically significant.

## 3. Results

### 3.1. *TP53*-Mutated BCs Exhibit Significantly Stronger Immune Signatures than* TP53*-Wildtype BCs

**Figure 1:**
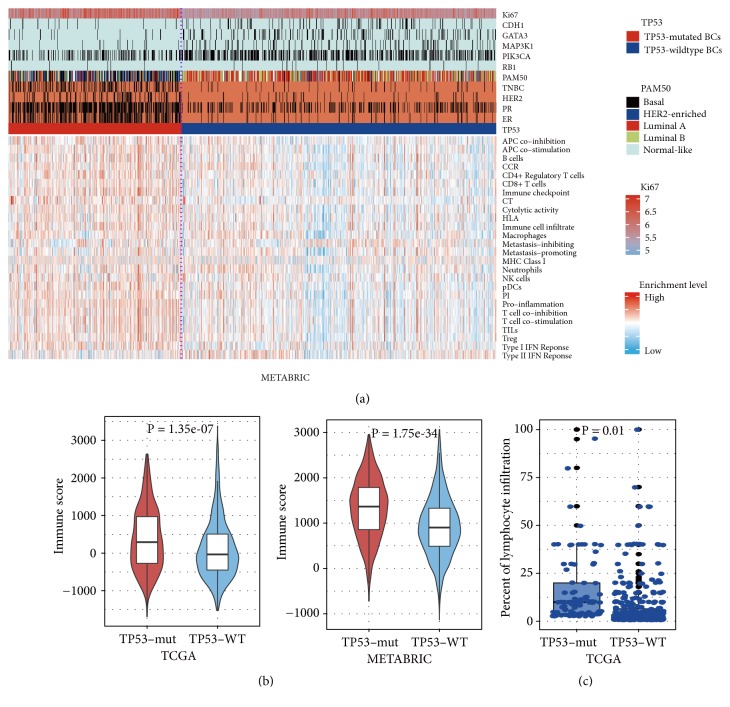
*TP53-mutated breast cancers (BCs) have increased immune activities compared to TP53-wildtype BCs*. (a) Heatmap showing the ssGSEA scores of 26 immune gene-sets in* TP53*-mutated and* TP53*-wildtype BCs (METABRIC). ssGSEA: single-sample gene-set enrichment analysis. TNBC: triple-negative breast cancer. Red color indicates higher enrichment levels (ssGSEA scores) of gene-sets, and blue color indicates lower enrichment levels of gene-sets in the heatmap.* RB1 *are more frequently mutated in* TP53*-mutated BCs while* CDH1*,* GATA3*,* MAP3K1*, and* PIK3CA *are more frequently mutated in* TP53*-wildtype BCs (Fisher's exact test, P<0.05). The black vertical lines in the horizontal bars beside gene symbols indicate that the genes are mutated in corresponding samples. The black vertical lines in the horizontal bar beside “TNBC” indicate that the sample is a TNBC. The black vertical lines in the horizontal bars beside “ER”, “PR,” and “HER2” indicate that the sample is ER-, PR-, or HER2-. (b)* TP53*-mutated BCs have significant higher degree of immune infiltration than* TP53*-wildtype cancers evaluated by ESTIMATE [29]. (c) The TCGA BC pathological slides data show that* TP53*-mutated BCs had markedly higher percent of lymphocyte infiltration than* TP53*-wildtype BCs.* TP53*-mut:* TP53*-mutated BCs.* TP53*-WT:* TP53*-wildtype BCs. It applies to all the other figures.

Strikingly, we found that almost all 26 immune signatures analyzed showed significantly higher enrichment levels in* TP53*-mutated BCs than in* TP53*-wildtype BCs consistently in both TCGA and METABRIC datasets (Mann-Whitney U test, P<0.05; Figure 1(a), Supplementary Figure S1A). Moreover,* TP53*-mutated BCs had significantly higher immune scores than* TP53*-wildtype BCs in both datasets (Mann-Whitney U test; P=1.35*∗*10^−7^. 1.75*∗*10^−34^ for TCGA and METABRIC, respectively) (Figure 1(b)). On the basis of the TCGA BC pathological slides data, we found that* TP53*-mutated BCs had markedly higher percentages of lymphocyte infiltration compared to* TP53*-wildtype BCs (Mann-Whitney U test, P=0.01) (Figure 1(c)). Altogether, these data indicate that* TP53* mutations are associated with elevated immune activity in BC.

Of the 15 immune cell type and function signatures [25], 14 showed significantly higher enrichment levels in* TP53*-mutated BCs than in* TP53*-wildtype BCs (Mann-Whitney U test, FDR<0.05) (Supplementary Table S5). Moreover, numerous marker genes of immune cells and function had significantly higher expression levels in* TP53*-mutated BCs than in* TP53*-wildtype BCs, e.g.,* CD8A* (CD8+ T cell),* B2M*,* HLA-A*, and* TAP1* (MHC Class I), and* GZMA* and* PRF1* (cytolytic activity) (Supplementary Table S5). The TILs signature composed of 122 genes [26] showed significantly higher enrichment levels in* TP53*-mutated BCs than in* TP53*-wildtype BCs (Mann-Whitney U test; P=2.84*∗*10^−8^, 4.22*∗*10^−33^ for TCGA and METABRIC, respectively) (Figure 1(a), Supplementary Figure S1B). Moreover, 112 (92%) TIL signature genes were more highly expressed in* TP53*-mutated BCs than in* TP53*-wildtype BCs in at least 1 dataset (88 in both datasets) (Supplementary Table S6; Figure S1C). Altogether, these data indicate that* TP53*-mutated BCs have higher degree of immune cell infiltration than* TP53*-wildtype BCs.

**Figure 2:**
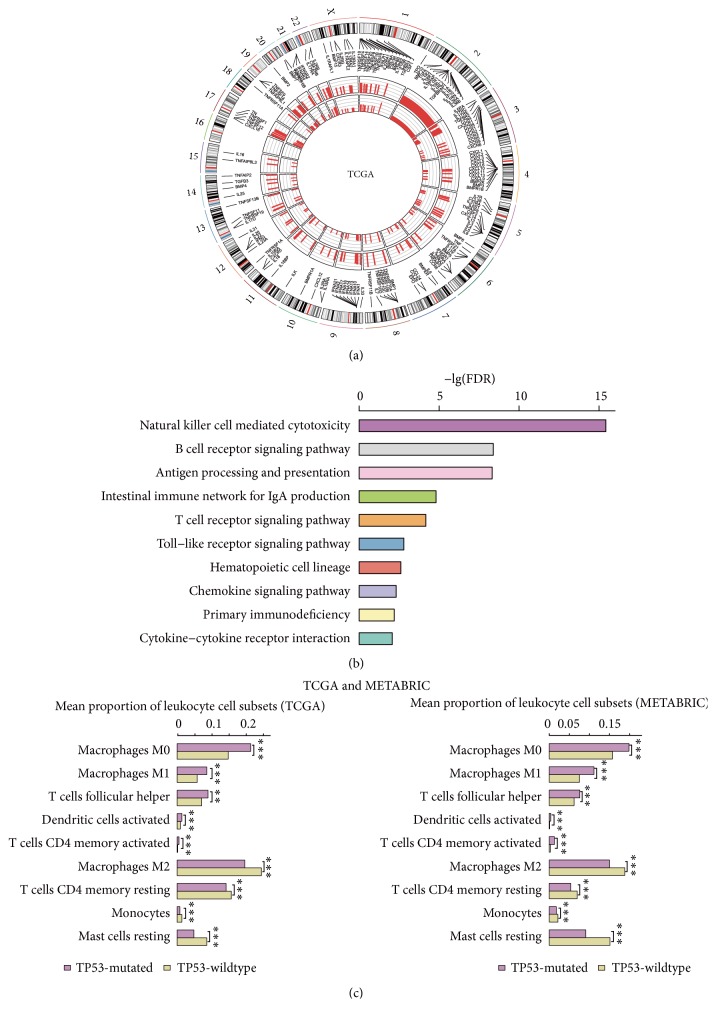
*TP53-mutated breast cancers (BCs) have higher activity of cytokines, immune pathways, and immune-promoting leukocyte cell subsets than TP53-wildtype BCs*. (a) Cytokine and cytokine receptor (CCR) genes show more frequent somatic copy number alterations (SCNAs) in* TP53*-mutated BCs than in* TP53*-wildtype BCs (Fisher's exact test, FDR<0.05). The outmost circle indicates 23 human chromosomes. The bars in both inner circles (outside and inside) indicate the frequency of SCNAs of CCR genes in* TP53*-mutated and* TP53*-wildtype BCs, respectively. A longer bar indicates a higher frequency of SCNAs. (b) Immune-related KEGG pathways upregulated in* TP53*-mutated BCs relative to* TP53*-wildtype BCs (FDR q-value<0.05). (c)* TP53*-mutated breast cancers (BCs) have significantly different leukocyte cell subset infiltrates estimated by CIBERSORT [30] compared to* TP53*-wildtype BCs.

Cytokines are important constituents of the immune system and play crucial roles in the immune regulation of cancer [42]. The enrichment levels of the CCR signature [29] were significantly higher in* TP53*-mutated BCs than in* TP53*-wildtype BCs in both datasets (Mann-Whitney U test; P=1.9*∗*10^−10^, 5.32*∗*10^−39^ for TCGA and METABRIC, respectively) (Figure 1(a); Supplementary Figure S1D; Table S7). Of the 261 CCR genes, 158 (61%) were more highly expressed in* TP53*-mutated BCs and 230 (88%) showed more frequent SCNAs in* TP53*-mutated BCs compared to* TP53*-wildtype BCs (Fisher's exact test, FDR < 0.05; Figure 2(a)). These data suggest that* TP53* mutations are associated with higher cytokine activity in BC.

The proinflammatory signature was more enriched in* TP53*-mutated BCs than in* TP53*-wildtype BCs in both datasets (Mann-Whitney U test; P=2.07*∗*10^−17^, 1.81*∗*10^−61^ for TCGA and METABRIC, respectively). Strikingly, all 16 proinflammatory genes [27] were upregulated in* TP53*-mutated BCs relative to* TP53*-wildtype BCs in at least 1 dataset (13 in both datasets) (Supplementary Figure S1E, S1F; Table S8). Remarkably,* STAT1* (signal transducer and activator of transcription 1) was upregulated in* TP53*-mutated BCs compared to both* TP53*-wildtype BCs and normal tissue. This gene has been shown to interact with p53 [43] and enhance immunosuppression in BC [44]. Another proinflammatory gene* GZMB* (granzyme B) was upregulated in* TP53*-mutated BCs compared to both* TP53*-wildtype BCs and normal tissue. This gene and* GZMA* (one of the cytolytic activity marker genes upregulated in* TP53*-mutated BCs) are associated with immune cytolytic activity as their protein products are mainly secreted by NK cells and cytotoxic T lymphocytes [25]. These data suggest that* TP53* mutations may promote inflammatory and immune cytolytic activities in BC. In addition, we found that another inflammatory signature parainflammation (PI) [28], a low-grade inflammatory reaction associated with carcinogenesis [28], was more enriched in* TP53*-mutated BCs than in* TP53*-wildtype BCs (Mann-Whitney U test; P=1.03*∗*10^−9^, 5.03*∗*10^−37^ for TCGA and METABRIC, respectively) and was also more enriched in both* TP53*-mutated BCs and* TP53*-wildtype BCs than in normal tissue (Mann-Whitney U test; P=6.89*∗*10^−12^, 0.003 for* TP53*-mutated and* TP53*-wildtype BCs, respectively) (Supplementary Table S8). These observations are in line with a previous study showing that PI significantly correlated with the p53 status in cancer [28].

GSEA [37] identified significantly upregulated pathways in* TP53*-mutated BCs compared to* TP53*-wildtype BCs, many of which were immune-related, including natural killer cell mediated cytotoxicity, B cell receptor signaling, antigen processing and presentation, intestinal immune network for IgA production, T cell receptor signaling, toll-like receptor signaling, hematopoietic cell lineage, chemokine signaling, primary immunodeficiency, and cytokine-cytokine receptor interaction (Figure 2(b)). In contrast, only the cytokine-cytokine receptor interaction pathway was significantly enriched in* TP53*-wildtype BCs. These results again demonstrate that* TP53* mutations are associated with elevated immune activity in BC.

Furthermore, we compared the proportions of 22 human leukocyte cell subsets that were evaluated by CIBERSORT [38] between* TP53*-mutated and* TP53*-wildtype BCs. We found that* TP53*-mutated BCs harbored higher proportions of activated dendritic cells, M0 macrophages, M1 macrophages, activated T cells CD4 memory, and T cells follicular helper cell subsets (Mann-Whitney U test; FDR<0.05; Figure 2(c)). In contrast,* TP53*-wildtype BCs harbored higher proportions of resting dendritic cells, M2 macrophages, resting mast cells, monocytes, and resting T cells CD4 memory cell subsets (Mann-Whitney U test; FDR<0.05; Figure 2(c)). This further demonstrates that* TP53* mutations are associated with stronger immune activity in BC. Intriguingly, M1 macrophages that incite inflammation had higher proportions in* TP53*-mutated BCs than in* TP53*-wildtype BCs, while M2 macrophages that repress inflammation and encourage tissue repair had lower proportions in* TP53*-mutated BCs. It suggests that* TP53* mutations may promote inflammatory behavior and inhibit tissue repair in BC, thereby contributing to higher invasiveness of* TP53*-mutated BCs [45].

### 3.2. *TP53* Mutations Are Associated with Elevated HLA Activity in BC

**Figure 3:**
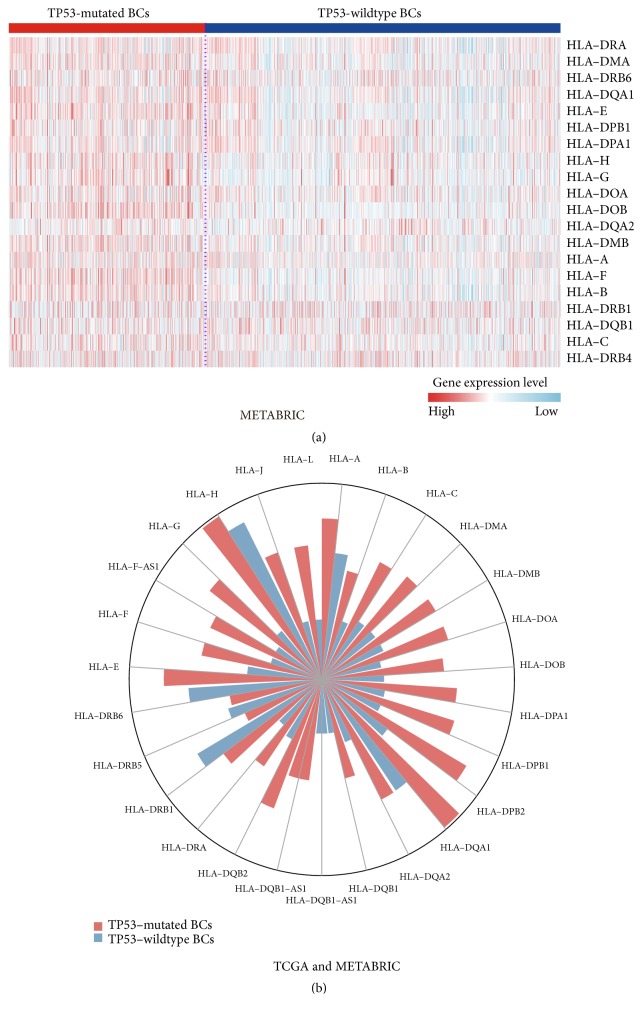
*TP53-mutated breast cancers (BCs) have more elevated expression of HLA genes than TP53-wildtype BCs*. (a) Heatmap shows that* TP53*-mutated BCs likely more highly express HLA genes than* TP53*-wildtype BCs (METABRIC). (b) HLA genes are more frequently amplified in* TP53*-mutated BCs than in* TP53*-wildtype BCs. The length of the bars in the rose diagram is proportional to the frequency of HLA gene amplification in* TP53*-mutated or* TP53*-wildtype BCs.

The products of HLA genes MHC proteins play important roles in the regulation of the immune system [46]. We found that most HLA genes showed significantly higher expression levels in* TP53*-mutated BCs than in* TP53*-wildtype BCs (Figure 3(a); Supplementary Table S9; Figure S1G). Moreover, HLA genes were more frequently amplified in* TP53*-mutated BCs compared to* TP53*-wildtype BCs (Figure 3(b)).* TP53*-mutated BCs had lower somatic mutation rates of HLA genes than* TP53*-wildtype BCs in TCGA (Fisher's exact test, P=0.02, OR=0.6), while METABRIC had no somatic mutation data available for HLA genes. These data suggest that* TP53* mutations may promote HLA activity in BC. This finding appears not to be consistent with a previous study showing that p53 increased expression of MHC proteins in cancer [47]. This inconsistency supports the notion that the p53 function is context-dependent and largely depends on the cell type [48, 49].

Gene mutations may yield neoantigens that are associated with antitumor immune response [11]. Although* TP53*-mutated BCs had markedly higher total mutation counts than* TP53*-wildtype BCs in TCGA (Mann-Whitney U test; P=4.18*∗*10^−25^), the numbers of gene mutations yielding predicted HLA-binding peptides [25] showed no significant differences between* TP53*-mutated and* TP53*-wildtype BCs (Mann-Whitney U test; P=0.4). It suggests that TMB or neoantigens may not be the essential factor explaining the differential immune activities between* TP53*-mutated and* TP53*-wildtype BCs.

### 3.3. Immune Activities Are Associated with Activities of p53-Regulated Pathways in BC

**Figure 4:**
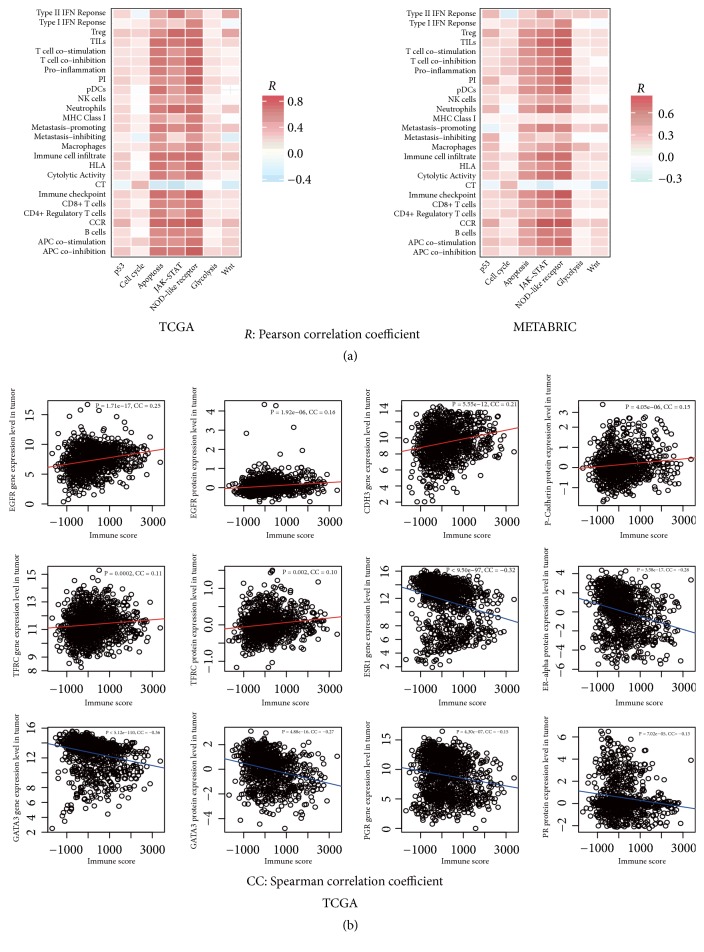
*Immune signatures significantly correlate with p53-regulated pathways, genes, and proteins in BC*. (a) Immune signatures likely positively correlate with the p53-mediated pathways that show higher activity in* TP53*-mutated BCs than in* TP53*-wildtype BCs. (b) Immune signatures positively correlate with* EGFR*,* CDH3*, and* TFRC*, and their protein products that are upregulated in* TP53*-mutated BCs, while they negatively correlate with* ESR1*,* GATA3*, and* PCR*, and their protein products that are downregulated in* TP53*-mutated BCs relative to* TP53*-wildtype BCs.

p53 plays important roles in regulating the cancer-associated pathways, e.g., cell cycle, apoptosis, DNA damage repair, autophagy, metabolism, inflammation, epithelial–mesenchymal transition (EMT), angiogenesis, and metastasis [48]. Accordingly,* TP53* mutations often result in the disturbance of the p53-mediated pathways [3]. Indeed, we found that a number of p53-mediated pathways showed significantly differential activity between* TP53*-mutated and* TP53*-wildtype BCs, such as the p53, cell cycle, apoptosis, Jak-STAT, NOD-like receptor, glycolysis, and Wnt pathways showing significantly higher activity in* TP53*-mutated BCs than in* TP53*-wildtype BCs (Mann-Whitney U test; P<0.05). Moreover, these pathways tended to positively correlate with the immune signatures analyzed (Figure 4(a)). These results indicate that the altered immune activity in* TP53*-mutated BCs could be associated with the disturbance of the p53-mediated pathways.

### 3.4. Identification of Genes and Proteins Differentially Expressed between* TP53*-Mutated and* TP53*-Wildtype BCs and Significantly Correlating with Immune Activity in BC

Based on the gene and protein expression data in TCGA, we identified the genes and proteins that were differentially expressed between* TP53*-mutated and* TP53*-wildtype BCs (Student's* t *test; FDR<0.05). Of these, 10 genes (*EGFR*,* CDH3*,* TFRC*,* CCNE1*,* CDK1*,* CDKN2A*,* CHEK1*,* FOXM1*,* NDRG1*, and* STMN1*) and their protein products had significantly higher expression levels in* TP53*-mutated BCs and 8 genes (*ESR1*,* GATA3*,* PGR*,* AR*,* ERBB3*,* BCL2*,* IGF1R*, and* CCND1*) and their protein products had significantly lower expression levels in* TP53*-mutated BCs. We termed the 10 genes and their protein products upregulated in* TP53*-mutated BCs as GPU and the 8 genes and their protein products downregulated in* TP53*-mutated BCs as GPD. Interestingly, GPU had a significant positive expression correlation with almost all 26 immune signatures and immune scores, while GPD had a significant negative expression correlation with them (Spearman correlation, FDR<0.05; Figure 4(b); Supplementary Figures S2A, S2B, S2C). These results showed that the expression of these molecules correlated with elevated or depressed immune activity in BC.

### 3.5. Association of Immune Activity with Clinical Outcomes in BC

**Figure 5:**
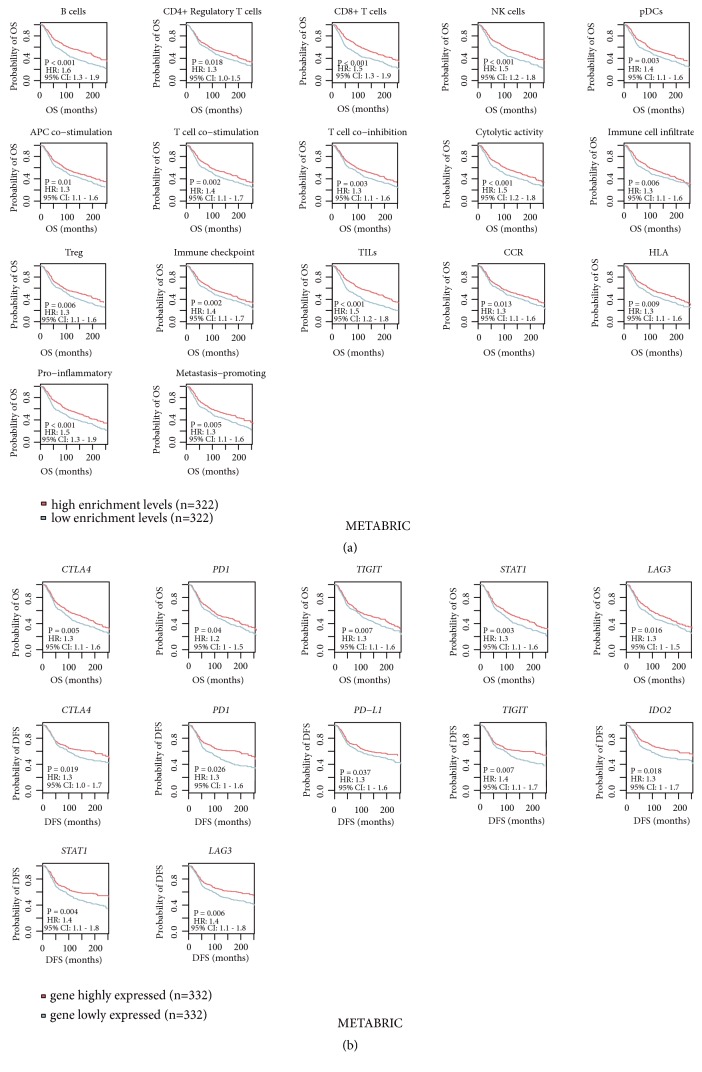
*Immune activities are positively associated with a 20-year survival prognosis in TP53-mutated BCs*. (a) Kaplan-Meier survival curves show that the elevated enrichment of immune signatures is associated with a better 20-year survival in* TP53*-mutated BCs. (b) Kaplan-Meier survival curves show that higher expression levels of immune genes are associated with a better 20-year survival in* TP53*-mutated BCs. The log-rank test P<0.05 indicates the significance of survival-time differences between two classes of patients. HR: hazard ratio; CI: confidence interval.

Among 26 immune signatures, 17 and 15 showed a significant correlation with survival (OS and/or DFS) prognosis in* TP53*-mutated and* TP53*-wildtype BCs, respectively (log-rank test; P*<*0.05) (Figure 5(a); Supplementary Figures S3A, S3B). Strikingly, elevated enrichment of the 17 immune signatures consistently correlated with a more favorable prognosis in* TP53*-mutated BCs. In contrast, 10 and 5 immune signatures were positively and negatively associated with survival in* TP53*-wildtype BCs, respectively. The B cell, cytolytic activity, T cell coinhibition, immune checkpoint, TILs, CCR, HLA, and proinflammatory signatures showed a positive correlation with survival consistently in both* TP53*-mutated and* TP53*-wildtype BCs. However, the CD4+ regulatory T cell signature showed a positive correlation with DFS in* TP53*-mutated BCs while showing a negative correlation in* TP53*-wildtype BCs (Supplementary Figures S3A, S3B; Table S10).

Furthermore, we found numerous immune-related genes whose expression was associated with survival prognosis in* TP53*-mutated and/or* TP53*-wildtype BCs. For example, the immune checkpoint genes* CTLA4*,* PD1*,* PD-L1*,* PD-L2*, and* TIGIT*, and the CD8+ T cell marker gene* CD8A *were positively associated with prognosis in both* TP53*-mutated and* TP53*-wildtype BCs (Figure 5(b); Supplementary Figure S3C). In addition, some genes were positively associated with prognosis exclusively in* TP53*-mutated or* TP53*-wildtype BCs, e.g.,* IL10*,* CD247*,* GZMA*,* GZMB*,* CD276*,* CCR4*, and* CCR7* (Supplementary Table S11). Interestingly, some genes had a positive correlation with survival in* TP53*-mutated BCs while having a negative correlation in* TP53*-wildtype BCs, e.g.,* IDO2*,* STAT1*, and* LAG3* (Figure 5(b); Supplementary Table S11; Figure S3C). The mechanism underlying these discrepancies may lie in that* TP53* mutations alter the tumor immune microenvironment (TIM) in BC.

### 3.6. In Vitro Experiments Validate that* TP53* Mutations Promote Immune Activity in BC

#### 3.6.1. *TP53* Mutations Increase the Expression of MHC Class I Genes in MCF-7 Cells

**Figure 6:**
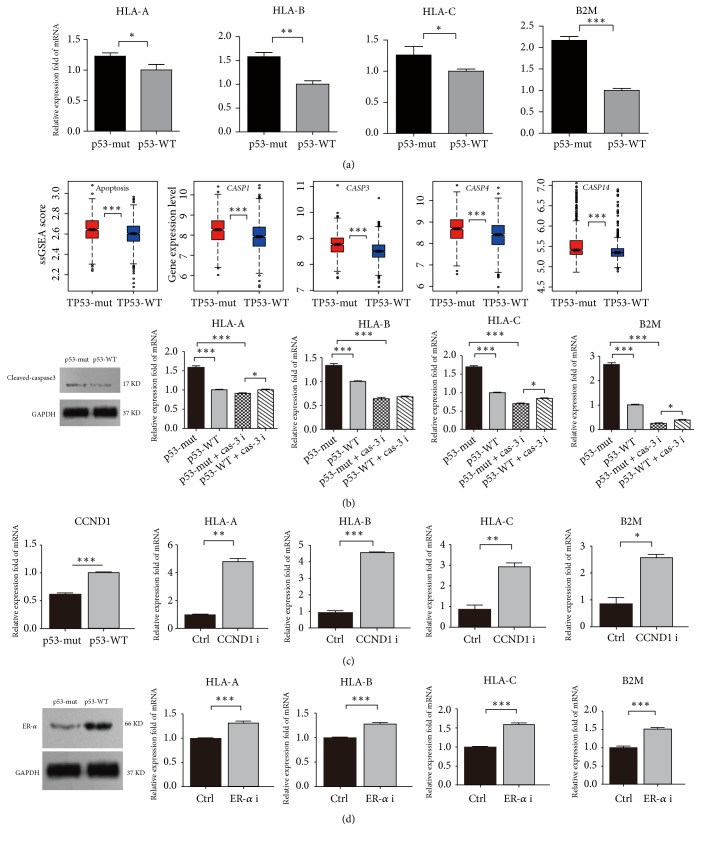
*The expression of MHC Class I genes is significantly upregulated in p53-mutant MCF-7 cells versus p53-wildtype MCF-7 cells and is regulated by the cell cycle, apoptosis, and estrogen receptor (ER) activities*. (a) MHC Class I genes (*HLA-A*,* HLA-B*,* HLA-C*, and* B2M*) have significantly higher mRNA expression levels in p53-mutant MCF-7 cells than in p53-wildtype MCF-7 cells, evident by real-time quantity PCR. (b) Promotion of apoptosis increases the expression of MHC Class I genes, evident by both computational and experimental analyses. (c) Inhibition of cell cycle increases the expression of MHC Class I genes. (d) Inhibition of ER alpha increases the expression of MHC Class I genes. p53-WT: p53-wildtype; p53-mut: p53-mutant; CCND1 i: CCND1 inhibitor; ERa i: ER alpha inhibitor.

We used a pair of isogenic BC cell lines with different p53 status (MCF-7 p53-wildtype versus MCF-7 p53-mutant) and evaluated MHC class I gene expression levels in both cell lines. The MHC Class I genes (*HLA-A*,* HLA-B*,* HLA-C*, and* B2M*) had significantly higher expression levels in p53-mutant MCF-7 cells than in p53-wildtype MCF-7 cells, demonstrated by real-time qPCR (Figure 6(a)). These experimental results verified that* TP53* mutations increased the expression of HLA molecules in BC.

#### 3.6.2. *TP53* Mutations Increase the Expression of MHC Class I Genes via Regulation of Apoptosis in BC

p53 plays an important role in regulation of apoptosis [50]. Surprisingly, our bioinformatics analysis showed that* TP53*-mutated BCs had significantly higher activity of the apoptosis pathway than* TP53*-wildtype BCs and that* TP53*-mutated BCs more highly expressed apoptosis-inducing caspases such as* CASP1*,* CASP3*,* CASP4*, and* CASP14* (Figure 6(b)). Furthermore, our experiments verified that caspase-3 expression markedly increased in p53-mutant MCF-7 cells versus p53-wildtype MCF-7 cells (Figure 6(b)). We treated both p53-mutant and p53-wildtype MCF-7 cells with the caspase-3 inhibitor Z-DEVD-FMK and found that the MHC Class I genes had markedly decreased expression in both p53-mutant and p53-wildtype MCF-7 cells (Figure 6(b)). Interestingly, we observed that p53-mutant MCF-7 cells more lowly expressed three out of the four MHC Class I genes than p53-wildtype MCF-7 cells after they were treated with Z-DEVD-FMK. These data indicate that apoptosis may have an appreciable effect on tumor immunity and that* TP53* mutations alter tumor immunity via regulation of apoptosis.

#### 3.6.3. *TP53* Mutations Increase the Expression of MHC Class I Genes via Regulation of Cell Cycle in BC

Our bioinformatics analysis showed that* CCND1 *(cyclin D1), a regulator of cyclin-dependent kinases, was downregulated in* TP53*-mutated BCs versus* TP53*-wildtype BCs. Furthermore, our experiments verified that* CCND1* had significantly lower mRNA expression levels in p53-mutant MCF-7 cells than in p53-wildtype MCF-7 cells (Figure 6(c)). We treated p53-wildtype MCF-7 cells with the cyclin D1 inhibitor abemaciclib and observed a substantial increase in the expression of MHC Class I genes in MCF-7 cells (Figure 6(c)). Thus, the alteration of the p53-mediated cell cycle pathway may contribute to the differential tumor immunity between* TP53*-mutated and* TP53*-wildtype BCs.

#### 3.6.4. *TP53* Mutations Increase the Expression of MHC Class I Genes via Downregulation of Estrogen Receptor Alpha

Our bioinformatics analysis showed that both* ESR1* and its protein product estrogen receptor alpha (ER*α*) were downregulated in* TP53*-mutated BCs versus* TP53*-wildtype BCs (Supplementary Table S12). This is consistent with previous studies showing that p53 upregulated ER*α* expression in BC and that* TP53* mutations downregulated ER*α* expression [51, 52]. Furthermore, our experiments verified that ER*α* was more lowly expressed in p53-mutant MCF-7 cells than in p53-wildtype MCF-7 cells (Figure 6(d)). We treated p53-wildtype MCF-7 cells with the ER*α* inhibitor MPP and observed a marked increase in the expression of MHC Class I genes in MCF-7 cells (Figure 6(d)). These results indicate that the downregulation of ER*α* may contribute to the elevated tumor immunity in* TP53*-mutated BCs.

#### 3.6.5. Mutant p53 Promotes Migration and Proliferation of NK Cells Cocultured with MCF-7 Cells

**Figure 7:**
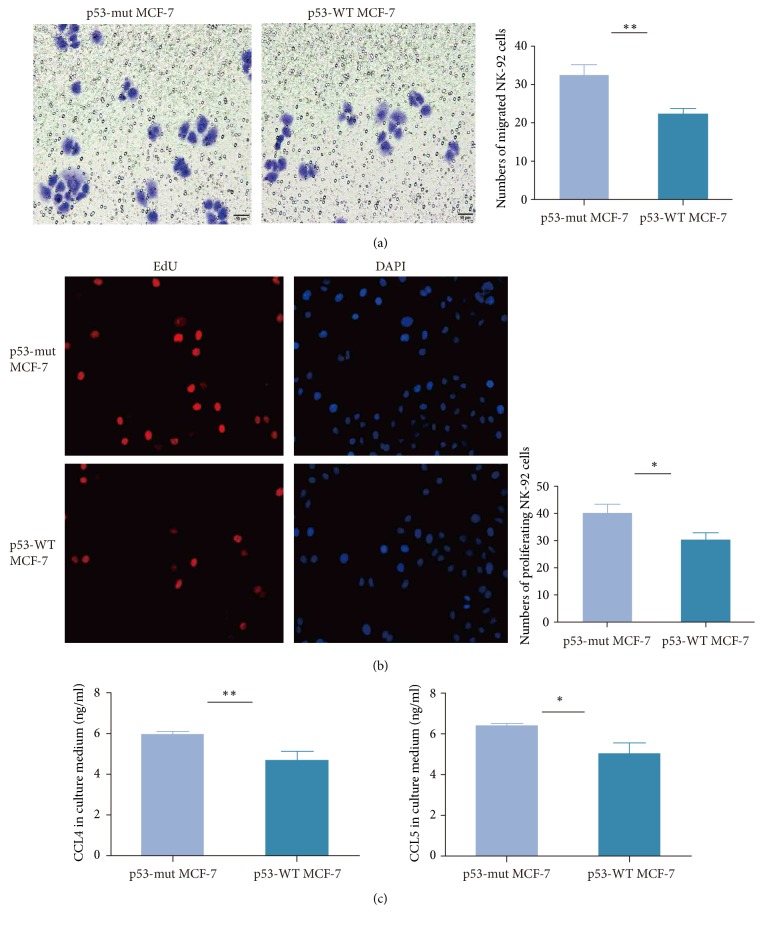
*Mutant p53 promotes migration and proliferation of NK cells cocultured with MCF-7 cells*. (a) NK92 cells cocultured with p53-mutant MCF-7 cells show stronger migration ability than NK92 cells cocultured with p53-wildtype MCF-7 cells, evident by transwell migration assay. (b) NK92 cells cocultured with p53-mutant MCF-7 cells show stronger proliferation ability than NK92 cells cocultured with p53-wildtype MCF-7 cells, evident by EdU proliferation assay. (c) Cytokines CCL4 and CCL5 have markedly higher levels in the serum containing NK92 cells cocultured with p53-mutant MCF-7 cells than in the serum containing NK92 cells cocultured with p53-wildtype MCF-7 cells, evident by quantitative enzyme-linked immunosorbent assay (ELISA).

We used the transwell migration and EdU proliferation assay to observe the migration and proliferation of NK92 cells cocultured with p53-mutant and p53-wildtype MCF-7 cells for 24h, respectively. We found that the number of migrated NK92 cells cocultured with p53-mutant MCF-7 cells far exceeded the number of migrated NK92 cells cocultured with p53-wildtype MCF-7 cells (Figure 7(a)). Moreover, the NK92 cells cocultured with p53-mutant MCF-7 cells showed significantly stronger proliferation ability compared to the NK92 cells cocultured with p53-wildtype MCF-7 cells (Figure 7(b)). Furthermore, we observed that the cytokines CCL4 and CCL5 had markedly higher levels in the serum containing NK92 cells cocultured with p53-mutant MCF-7 cells than in the serum containing NK92 cells cocultured with p53-wildtype MCF-7 cells (Figure 7(c)). These observations verified our computational results that* TP53*-mutated BCs had stronger activities of immune cells including NK cells and more highly expressed a number of CCR genes including* CCL4* and* CCL5* than* TP53*-mutated BCs. These findings are also consistent with previous studies showing that cytokines such as CCL4 could induce NK cells migration [53] and that activated NK cells could secrete cytokines to mediate immune response [54].

## 4. Discussion

We performed a comprehensive portrait of the association between* TP53* mutations and immune signatures in BC. We found that* TP53*-mutated BCs showed significantly higher levels of immune infiltration and higher activity of various immune cells, function, and pathways than* TP53*-wildtype BCs (Figure 1(a)).* TP53*-mutated BCs had higher proportions of activated immune cell subsets and lower proportions of resting immune cell subsets compared to* TP53*-wildtype BCs within the TME.* TP53*-mutated BCs have significant differences in clinical features compared to* TP53*-wildtype BCs. Typically,* TP53*-mutated BCs contain a higher proportion of ER-, PR-, HER2+, or triple-negative/basal-like BCs. Previous studies have shown that the ER- and HER2+ features were associated with stronger immunogenic activity in BC [22, 55]. Thus, both features may contribute to the higher immune activity in* TP53*-mutated BCs as compared to* TP53*-wildtype BCs. However, when comparing the enrichment levels of the 26 immune signatures between* TP53*-mutated and* TP53*-wildtype BCs within the ER+ subtype of BC, we obtained similar results that almost all these immune signatures were more enriched in* TP53*-mutated ER+ BCs than in* TP53*-wildtype ER+ BCs (Supplementary Table S13). Similarly,* TP53*-mutated HER2- BCs had significantly higher enrichment levels of immune signatures than* TP53*-wildtype HER2- BCs (Supplementary Table S14). These results indicate that the* TP53* mutation itself is capable of contributing to the elevated immune activity in BC as was further verified by in vitro experiments. Our computational and experimental results suggest that* TP53* mutations may alter immune activity in BC via regulation of the p53-mediated pathways, including cell cycle, apoptosis, Wnt, Jak-STAT, NOD-like receptor, and glycolysis. It should be noted that previous studies have demonstrated that p53 could increase immune activity in various cancers, e.g., colon cancer [47], gastric cancer [56], and lymphoma [8], as appears not to be in line with the present findings. Nevertheless, a number of studies have shown that p53 functions in a context-dependent fashion [8, 48, 49]. Thus, these distinct effects of p53 in regulating tumor immunity could be attributed to the different cellular contexts. Numerous studies have shown that* TP53* mutations may result in not only loss of the wildtype p53 tumor-suppressive function but also gain of oncogenic function of mutant p53 [57]. A recent study showed that* TP53* gain of function mutation could promote inflammatory activity in glioblastoma [58]. To explore whether the elevated immune/inflammatory activity in* TP53*-mutated BC is attributed to* TP53* gain of function mutations, we silenced* TP53* expression in MCF-7 cells by siRNA. Interestingly, we observed a significant increase in the expression levels of MHC Class I genes in p53-knockdown MCF-7 cells compared with p53-wildtype MCF-7 cells (Supplementary Figure S5). This indicates that the elevated immune/inflammatory activity in* TP53*-mutated BC is likely caused by* TP53* loss of function mutations.

One tumor sample may contain a certain percentage of nontumor cells such as normal cells and stromal cells. To exclude the impact of nontumor associated cells on the present results, we selected the BC samples composed of 100% tumor cells based on the TCGA BC pathological slides data. We observed the similar results that almost all 26 immune signatures exhibited significantly higher enrichment levels in the* TP53*-mutated class than in the* TP53*-wildtype class of these samples (Mann-Whitney U test; FDR<0.05) (Supplementary Table S15). Thus, the differential immune activity between* TP53*-mutated and* TP53*-wildtype BCs referred to the actual difference in tumor immunity.

Cancer-testis (CT) antigens are a group of immunogenic proteins overexpressed in many cancers [59]. We found that the CT antigen signature [30] was more active in* TP53*-mutated BCs than in* TP53*-wildtype BCs in both datasets (Mann-Whitney U test; P=9.52*∗*10^−35^, 9.46*∗*10^−24^ for TCGA and METABRIC, respectively) (Figure 1(a); Supplementary Figure S4A). Many CT antigen genes were upregulated in* TP53*-mutated BCs and encode the CT antigens that are potential targets for developing cancer vaccines, e.g.,* MAGEA*,* NY-ESO-1*, and* PRAME* (Supplementary Figure S4B; Table S16). It suggests that p53 could inhibit the expression of many CT antigens, a finding in line with a prior study [60].

**Figure 8:**
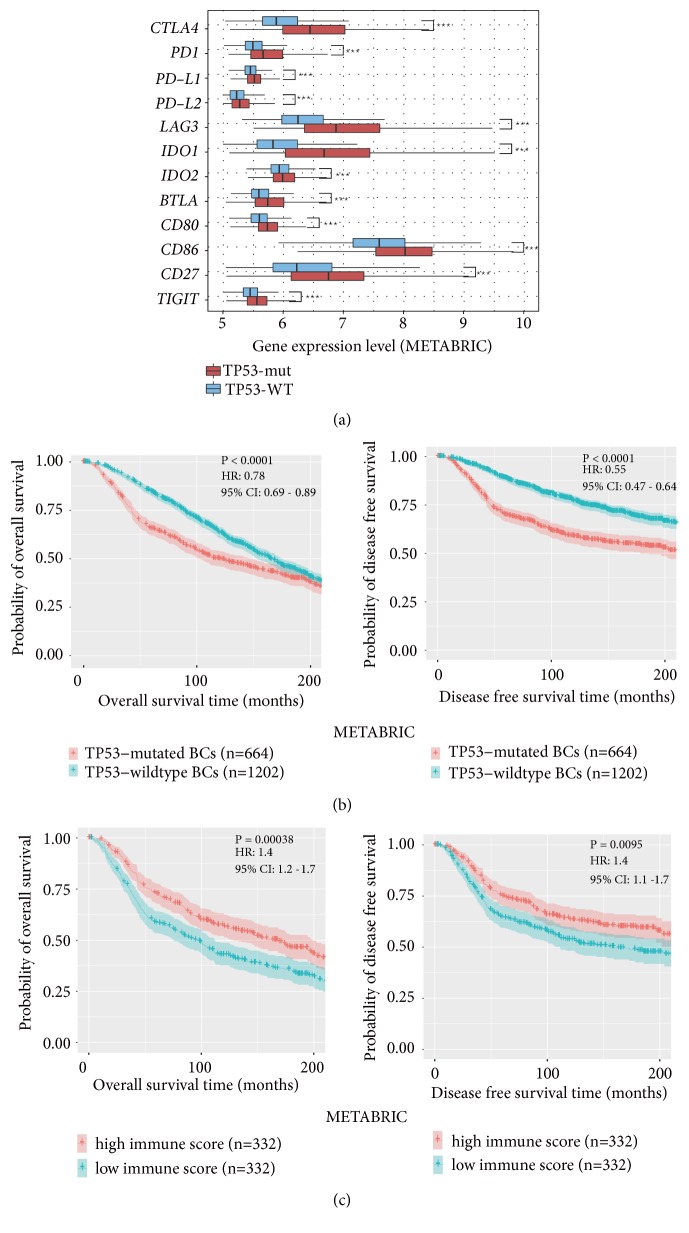
*TP53-mutated breast cancers (BCs) more highly express immune checkpoint genes and have a worse 20-year survival than TP53-wildtype BCs and associated with unfavorable survival prognosis in BC, while higher degree of immune cell infiltration is associated with a 20-year better survival prognosis in BC*. (a) A number of important immune checkpoint genes are upregulated in* TP53*-mutated BCs versus* TP53*-wildtype BCs. (b) Kaplan-Meier survival curves show that* TP53*-mutated BCs have a worse 20-year survival prognosis than* TP53*-wildtype BCs. (c) Kaplan-Meier survival curves show that higher degree of immune cell infiltration is associated with a better 20-year survival prognosis in* TP53*-mutated BCs. The log-rank test P<0.05 indicates the significance of survival-time differences between two classes of patients.

Interestingly, we found that* TP53*-mutated BCs had remarkably higher enrichment levels of Treg signature and immune checkpoint signature [31] than* TP53*-wildtype BCs in both datasets (Mann-Whitney U test; P<10^−10^) (Figure 1(a); Supplementary Figure S4C; Table S17). In particular, numerous notable immune checkpoint genes were upregulated in* TP53*-mutated BCs, including* CTLA4*,* PD1*,* PD-L1*,* PD-L2*,* LAG3*,* IDO1/2*,* BTLA*,* CD80*,* CD8*6,* CD27*, and* TIGIT* (Figure 8(a); Supplementary Table S18; Figure S4D). These results suggest that p53 may play a role in inhibiting tumor immunosuppression in BC. Weyden et al. [32] identified 19 genes which function in immune regulation of cancer metastasis, of which 12 promoted tumor metastasis and 7 inhibited tumor metastasis. The enrichment levels of the metastasis-promoting signature were markedly higher in* TP53*-mutated BCs than in* TP53*-wildtype BCs in both datasets (Mann-Whitney U test; P=3.62*∗*10^−6^, 0.003 for TCGA and METABRIC, respectively) (Supplementary Figure 4E). In contrast, the metastasis-inhibiting signature exhibited significantly lower enrichment levels in* TP53*-mutated BCs than in* TP53*-wildtype BCs in TCGA (Mann-Whitney U test; P=0.01) (Supplementary Figure 4E). Notably,* SPNS2 *(sphingolipid transporter 2) which most incited tumor metastasis by regulating lymphocyte trafficking [32], had higher expression levels in* TP53*-mutated BCs than in* TP53*-wildtype BCs in TCGA (Student's* t* test; FDR=1.34*∗*10^−6^;* SPNS2* expression data was lacking in METABRIC). These results suggest that* TP53*-mutated BCs are metastasis-prone and that this characteristic may be attributed to the defect in p53 immune regulation of BC and its TME.

The elevated expression of immunosuppressive, proinflammatory, and metastasis-promoting signatures in* TP53*-mutated BCs may promote tumor invasion and lead to a worse prognosis in BC. Indeed, previous studies have shown that p53 mutations were associated with unfavorable clinical outcomes in BC [61, 62]. The METABRIC data also showed that* TP53*-mutated BCs had worse OS and DFS compared to* TP53*-wildtype BCs (Figure 8(b)). Moreover,* TP53*-mutated BCs more highly expressed Ki67 (a marker for cell proliferation) than* TP53*-wildtype BCs (Figure 1(a)), again indicating the higher aggressiveness of* TP53*-mutated BCs. Interestingly, the activities of different immune cell types, function, and pathways, and the immune cell infiltration degree were consistently positively associated with survival prognosis in* TP53*-mutated BCs (Figures 5(a), 5(b), and 8(c)). It is sensible that the elevated enrichment of CD8+ T cell, B cell, NK cell, cytolytic activity, HLA, immune cell infiltrate, TILs, and CCR is associated with favorable clinical outcomes in cancer since these immune signatures can promote anticancer immune response. Furthermore, the observation that the elevated enrichment of Treg, immune checkpoint, proinflammatory, and metastasis-promoting immune signatures was associated with better survival in* TP53*-mutated BCs may be due to the fact that the elevated immunosuppressive activity is likely to promote chemotherapy sensitivity of* TP53*-mutated BCs [63]. Thus, to achieve successes in immunotherapy of* TP53*-mutated BCs, the effective combination of chemotherapy with immunotherapy may represent a promising direction [64].

Interestingly, compared to* TP53*-wildtype BCs,* TP53*-mutated BCs more highly express a majority of the gene targets for immunotherapy agents that are currently used in the clinic or clinical trials [65] (Supplementary Table S19). It indicates that these immunotherapy agents may be more effective against* TP53*-mutated BCs than* TP53*-wildtype BCs. In fact, several clinical trials [20, 21] have shown that immune checkpoint blockade was effective against TNBC, a BC subtype with a high* TP53* mutation rate.

## 5. Conclusions

*TP53 *mutations promote immune activity in BC. This finding suggests that the* TP53* mutation status could be a useful biomarker for stratifying BC patients responsive to immunotherapy.

## Data Availability

The TCGA data can be downloaded from the genomic data commons data portal (https://portal.gdc.cancer.gov/), and the METABRIC data can be downloaded from cBioPortal (http://www.cbioportal.org).

## Supplementary Materials

Supplementary Materials
*Supplementary Tables*. Table S1: the list of 26 immune signatures and related gene sets. Table S2: sample sizes of breast cancers. Table S3: ssGSEA scores of immune signature in TCGA and METABRIC. Table S4: primer sequences used for real time quantity PCR. Table S5: comparison of the enrichment levels of 15 immune cell types and function signatures between two classes of samples. Table S6: comparison of the enrichment levels of the tumor-infiltrating lymphocytes signature between two classes of samples. Table S7: comparison of the enrichment levels of the cytokine and cytokine receptor signature between two classes of samples. Table S8: comparison of the enrichment levels of the inflammation-promoting and parainflammation (PI) signatures between two classes of samples. Table S9: comparison of the enrichment levels of the HLA signature between two classes of samples. Table S10: comparisons of the ssGSEA scores of immune signatures between* TP53*-mutated and* TP53*-wildtype BCs and their associations with survival prognosis in BC. Table S11: comparisons of the expression levels of immune genes between* TP53*-mutated and* TP53*-wildtype BCs and their associations with survival prognosis in BC. Table S12: comparisons of the expression levels of genes and their protein products between TP53-mutated and TP53-wildtyped BCs. Table S13: comparisons of the enrichment levels of immune signatures between* TP53*-mutated and* TP53*-wildtype BCs within the ER+ subtype of BC. Table S14: comparisons of the enrichment levels of immune signatures between* TP53*-mutated and* TP53*-wildtype BCs within the HER2- subtype of BC. Table S15: comparisons of the enrichment levels of immune signatures between* TP53*-mutated and* TP53*-wildtype BCs within the 100% tumor purity of BC. Table S16: comparisons of the enrichment levels of the cancer-testis signature between two classes of samples. Table S17: comparisons of the enrichment levels of the Treg signature between two classes of samples. Table S18: comparisons of the enrichment levels of the immune checkpoint signature between two classes of samples. Table S19: comparisons of the expression levels of the genes targeted by immunotherapy agents in clinical use or trials or in preclinical development between* TP53*-mutated and* TP53*-wildtype BCs.* Supplementary Figures Legends*. Figure S1:* TP53-mutated breast cancers (BCs) have increased immune activity compared to TP53-wildtype BCs*. A: heatmap shows the ssGSEA scores of 26 immune signatures in* TP53*-mutated and in* TP53*-wildtype BCs (TCGA). ssGSEA: single-sample gene-set enrichment analysis; TNBC: triple-negative breast cancer.* RB1* is more frequently mutated in* TP53*-mutated BCs while* CDH1*,* GATA3*,* MAP3K1*, and* PIK3CA* are more frequently mutated in* TP53*-wildtype BCs (Fisher's exact test; P<0.05). The black vertical lines in the horizontal bars beside gene symbols indicate that the genes are mutated in corresponding samples. The black vertical lines in the horizontal bar beside “TNBC” indicate that the samples are TNBC. The black vertical lines in the bars beside “ER”, “PR,” and “HER2” indicate that the samples are ER-, PR-, or HER2-. B:* TP53*-mutated BCs have higher levels of tumor-infiltrating lymphocytes (TILs) infiltration than* TP53*-wildtype BCs (Mann-Whitney U test; p<0.001). C: heatmaps show that* TP53*-mutated BCs likely more highly express TILs genes than* TP53*-wildtype BCs. D: TP53-mutated BCs have higher enrichment levels of the cytokine and cytokine receptor (CCR) signature than* TP53*-wildtype BCs. E: heatmaps show that* TP53*-mutated BCs likely more highly express proinflammatory and parainflammation (PI) gene signatures than* TP53*-wildtype BCs. F: most of the proinflammatory genes are upregulated in* TP53*-mutated BCs relative to* TP53*-wildtype BCs. G: heatmaps show that* TP53*-mutated BCs likely more highly express HLA genes than* TP53*-wildtype BCs in TCGA. *∗*, P<0.05. *∗∗*, P<0.01. *∗∗∗*, P< 0.001. It also applies to the following figures. Figure S2:* the genes and their protein products have a significant expression correlation with immune activities in BC that are differentially expressed between TP53-mutated and TP53-wildtype breast cancers (BCs)*. A: the genes* TFRC*,* CDH3*, and* EGFR* show a positive expression correlation with immune activities in BC. B: the genes* GATA3*,* IGF1R*,* PGR*,* ESR1*,* ERBB3*,* BCL2*, and* AR* show a negative expression correlation with immune activities in BC. C:* AR*,* ERBB3*,* BCL2*, and* IGF1R*, and their protein products consistently show a negative expression correlation with immune scores in BC. Figure S3:* association of immune signatures with clinical outcomes in breast cancers (BCs)*. A: Kaplan-Meier survival curves show that the upregulation of immune signatures is consistently associated with better survival in* TP53*-mutated BCs. B: Kaplan-Meier survival curves show that the upregulation of immune signatures is associated with better or worse survival in* TP53*-wildtype BCs. C: the genes* IDO2*,* STAT1*, and* LAG3* have a negative expression correlation with survival prognosis in* TP53*-wildtype BCs. Figure S4:* TP53-mutated breast cancers (BCs) have higher expression levels of cancer-testis (CT) antigens, Treg, immune checkpoint, metastasis-promoting, and metastasis-inhibiting immune signatures than TP53-wildtype BCs*. A: a number of genes encoding the CT antigens that are potential targets for developing cancer vaccines are upregulated in* TP53*-mutated BCs relative to* TP53*-wildtype BCs. B: TP53-mutated BCs have higher enrichment levels of the CT antigen signature than* TP53*-wildtype BCs. C:* TP53*-mutated BCs have higher enrichment levels of the Treg and immune checkpoint signatures than* TP53*-wildtype BCs. D: a number of important immune checkpoint genes are more highly expressed in* TP53*-mutated BCs than in* TP53*-wildtype BCs in TCGA. Treg: regulatory T cell. E: the metastasis-promoting and the metastasis-inhibiting immune signatures are significantly upregulated and downregulated in* TP53*-mutated BCs versus* TP53*-wildtype BCs. Figure S5:* knockdown of p53 by siRNA significantly increases the expression of MHC Class I genes in MCF-7 cells*.
