# Supplementary material for: TP53 Mutations Promote Immunogenic Activity in Breast Cancer
Source: J Oncol. 2019 Jun 2;2019:5952836. doi: 10.1155/2019/5952836 (PMC6582869; doi:10.1155/2019/5952836)
Supplement: Supplementary Materials — Supplementary Tables. Table S1: the list of 26 immune signatures and related gene sets. Table S2: sample sizes of breast cancers. Table S3: ssGSEA scores of immune signature in TCGA and METABRIC. Table S4: primer sequences used for real time quantity PCR. Table S5: comparison of the enrichment levels of 15 immune cell types and function signatures between two classes of samples. Table S6: comparison of the enrichment levels of the tumor-infiltrating lymphocytes signature between two classes of samples. Table S7: comparison of the enrichment levels of the cytokine and cytokine receptor signature between two classes of samples. Table S8: comparison of the enrichment levels of the inflammation-promoting and parainflammation (PI) signatures between two classes of samples. Table S9: comparison of the enrichment levels of the HLA signature between two classes of samples. Table S10: comparisons of the ssGSEA scores of immune signatures between TP53-mutated and TP53-wildtype BCs and their associations with survival prognosis in BC. Table S11: comparisons of the expression levels of immune genes between TP53-mutated and TP53-wildtype BCs and their associations with survival prognosis in BC. Table S12: comparisons of the expression levels of genes and their protein products between TP53-mutated and TP53-wildtyped BCs. Table S13: comparisons of the enrichment levels of immune signatures between TP53-mutated and TP53-wildtype BCs within the ER+ subtype of BC. Table S14: comparisons of the enrichment levels of immune signatures between TP53-mutated and TP53-wildtype BCs within the HER2- subtype of BC. Table S15: comparisons of the enrichment levels of immune signatures between TP53-mutated and TP53-wildtype BCs within the 100% tumor purity of BC. Table S16: comparisons of the enrichment levels of the cancer-testis signature between two classes of samples. Table S17: comparisons of the enrichment levels of the Treg signature between two classes of samples. Table S18: comparisons [file 5952836.f1.zip › 5952836.f1/Supplementary Tables 10-11.docx]

**Table S10. Comparisons of the ssGSEA scores of immune signatures between *TP53*-mutated and *TP53*-wildtype BCs, and their associations with survival prognosis in BC.**

| **Immune gene-sets** | | ***TP53*-mutated BCs vs.**  ***TP53*-wildtype BCs**^a^ | ***TP53*-mutated BCs vs. normal tissue** ^b^ | ***TP53*-wildtype BCs vs. normal tissue**^c^ | **Association of ssGSEA scores with survival in *TP53*-mutated BCs**^d^ | **Association of ssGSEA scores with survival in *TP53*-wildtype BCs**^d^ |
| --- | --- | --- | --- | --- | --- | --- |
| 15 immune cell types and function | B cell | up (1) | NS | NS | OS (M), DFS (M) | OS (M), |
|  | CD4+ regulatory T cell | up | up | up | OS (M), DFS (M) | DFS (M) |
|  | CD8+ T cell | up | up | NS | OS (M), DFS (M) | NS |
|  | NK cell | up | down | down | OS (M), DFS (M) | NS |
|  | cytolytic activity | up | up | NS | OS (M), DFS (M) | OS (M, T) |
|  | macrophages | up | up | up | NS | NS |
|  | MHC class I | up | up | up | NS | NS |
|  | APC co-stimulation | up | up | up | OS (M) | NS |
|  | T cell co-stimulation | up | up | up | OS (M), DFS (M) | NS |
|  | APC co-inhibition | up | down | down | NS | NS |
|  | T cell co-inhibition | up | up | down | OS (M), DFS (M) | OS (T) |
|  | neutrophils | up (1) | down | NS | NS | NS |
|  | pDCs | up | up | up | OS (M), DFS (M) |  |
|  | Type I IFN reponse | up | up | up | NS | OS (M), DFS (M) |
|  | Type II IFN reponse | down | down | down | NS | OS (M), DFS (M) |
| immune cell infiltrate | | up | up | NS | OS (M), DFS (M) | OS (T), DFS (M) |
| Treg | | up | NS | down | OS (M), DFS (M) | NS |
| immune checkpoint | | up | up | NS | OS (M), DFS (M) | OS (T) |
| TILs | | up | up | NS | OS (M), DFS (M) | OS (T) |
| CCR | | up | down | down | OS (M) | OS (M, T), |
| CT | | up | up | up | NS | OS (M), DFS (M, T) |
| HLA | | up | up | up | OS (M), DFS (M) | OS (T) |
| pro-inflammatory | | up | up | up | OS (M), DFS (M) | OS (T) |
| parainflammation | | up | up | up | NS | DFS (M) |
| metastasis-promoting | | up | up | NS | OS (M), DFS (M) | NS |
| metastasis-inhibiting | | down (1) | up | up | NS | OS (T) |

^a^ The "up" indicates that *TP53*-mutated BCshad higher ssGSEA scores for the gene-set than *TP53*-wildtype BCs found in both datasets, and the "up (1)" indicates found in only one dataset; The "down" indicates that *TP53*-mutated BCshad lower ssGSEA scores for the gene-set than *TP53*-wildtype BCs found in both datasets, and the "down (1)" indicates found in only one dataset.

^b^ The "up" and "down" indicates that *TP53*-mutated BCshad higher and lower ssGSEA scores for the gene-set than normal tissue, respectively, and the "NS" indicates that the ssGSEA scores for the gene-set were not statistically different between *TP53*-mutated BCs and normal tissue.

^c^ The "up" and "down" indicates that *TP53*-wildtype BCshad higher and lower ssGSEA scores for the gene-set than normal tissue, respectively, and the "NS" indicates that the ssGSEA scores for the gene-set were not statistically different between *TP53*-wildtype BCs and normal tissue.

^d^ OS: Increased ssGSEA scores were associated with better overall survival prognosis in cancers.

DFS: Increased ssGSEA scores were associated with better disease-free survival prognosis in cancers.

OS: Increased ssGSEA scores were associated with worse overall survival prognosis in cancers.

DFS: Increased ssGSEA scores were associated with worse disease-free survival prognosis in cancers.

"(M)", "(T)", and "(M, T)" indicate that the association of ssGSEA scores with survivalprognosis in cancers was significant in METABRIC, TCGA, and both METABRIC and TCGA, respectively.

**Table S11. Comparisons of the expression levels of immune genes between *TP53*-mutated and *TP53*-wildtype BCs, and their associations with survival prognosis in BC.**

| **Immune gene** | ***TP53*-mutated BCs vs.**  ***TP53*-wildtype BCs**^a^ | ***TP53*-mutated BCs vs. normal tissue** ^b^ | ***TP53*-wildtype BCs vs. normal tissue**^c^ | **Association of expression levels with survival in *TP53*-mutated BCs**^d^ | **Association of expression levels with survival in *TP53*-wildtype BCs**^d^ |
| --- | --- | --- | --- | --- | --- |
| PDCD1 | up | up | up | OS (M), DFS (M) | OS (T) |
| CTLA4 | up | up | up | OS (M), DFS (M) | OS (T) |
| PD-L1 | up | NS | down | DFS (M, T) | OS (T) |
| PD-L2 | up | down | down | NS | NS |
| CCR1 | up | up | NS | OS (M), DFS (M) | DFS(T) |
| CCR4 | up | up | up | OS (M), DFS (M) | NS |
| CCR7 | up | up | up | OS (M), DFS (M) | NS |
| IRF1 | up | up | up | OS (M), DFS (M) | OS (T), DFS (T) |
| CD28 | up | NS | down | NS | OS (T) |
| PRF1 | up | NS | down | OS (M) | OS (T) |
| HLA-B | up | up | up | OS (M), DFS (M) | NS |
| HLA-E | up | down | down | NS | OS (M), DFS (M) |
| HLA-F | up | up | NS | OS (M), DFS (M) | OS (T) |
| HLA-G | up | up | NS | OS (M), DFS (M) | NS |
| HLA-H | up | up | up | OS (M) | OS (T) |
| TIGIT | up | up | up | OS (M), DFS (M) | OS (T) |
| *SELL* | up | up | up | OS (M), DFS (M) | OS (T), DFS (T) |
| CD27 | up | up | NS | OS (M), DFS (M) | OS (T) |
| CD40 | up | down | down | OS (M) | OS (T) |
| CD47 | up | down | down | NS | NS |
| CD96 | up (1) | NS | down | NS | NS |
| CD276 | up | up | up | DFS (M) | NS |
| DOCK11 | up | down | down | OS (M) | OS (M) |
| *CD8A* | up | NS | NS | OS (M) | OS (M, T), DFS (M) |
| *IDO1* | up | up | NS | DFS (M) | OS (M, T), DFS (M) |
| *IDO2* | up | up | up | OS (M), DFS (M) | OS (T) , DFS (M) |
| *GZMA* | up | up | NS | NS | OS (M, T), DFS (M) |
| *IL10* | up (1) | up | NS | NS | OS (M), DFS (M) |
| *IL11* | up | up | up | NS | DFS (T) |
| *IL15* | up | NS | down | DFS (M) | OS (M, T), DFS (M) |
| *CD247* | up | NS | down | NS | OS (M, T), DFS (M) |
| *GZMB* | up | up | NS | NS | OS (M), DFS (M) |
| *STAT1* | up | up | up | OS (M), DFS (M) | OS (M), DFS (M) |
| *STAT4* | up | down | down | OS (M) | OS (M, T), DFS (M) |
| ***FOXP3*** | up (1) | up | up | DFS (M) | NS |
| CD79A | up | NS | NS | OS (M), DFS (M) | OS (M,T) |
| CYBB | up | NS | down | NS | NS |
| CCL2 | up | down | down | NS | DFS (T) |
| *LAG3* | up | up | up | OS (M), DFS (M) | OS (M), DFS (M) |
| VTCN1 | up | NS | down | NS | OS (M), DFS (M,T) |
