# Supplementary figures and images for: TP53 Mutations Promote Immunogenic Activity in Breast Cancer
Source: J Oncol. 2019 Jun 2;2019:5952836. doi: 10.1155/2019/5952836 (PMC6582869; doi:10.1155/2019/5952836)

Figure S1

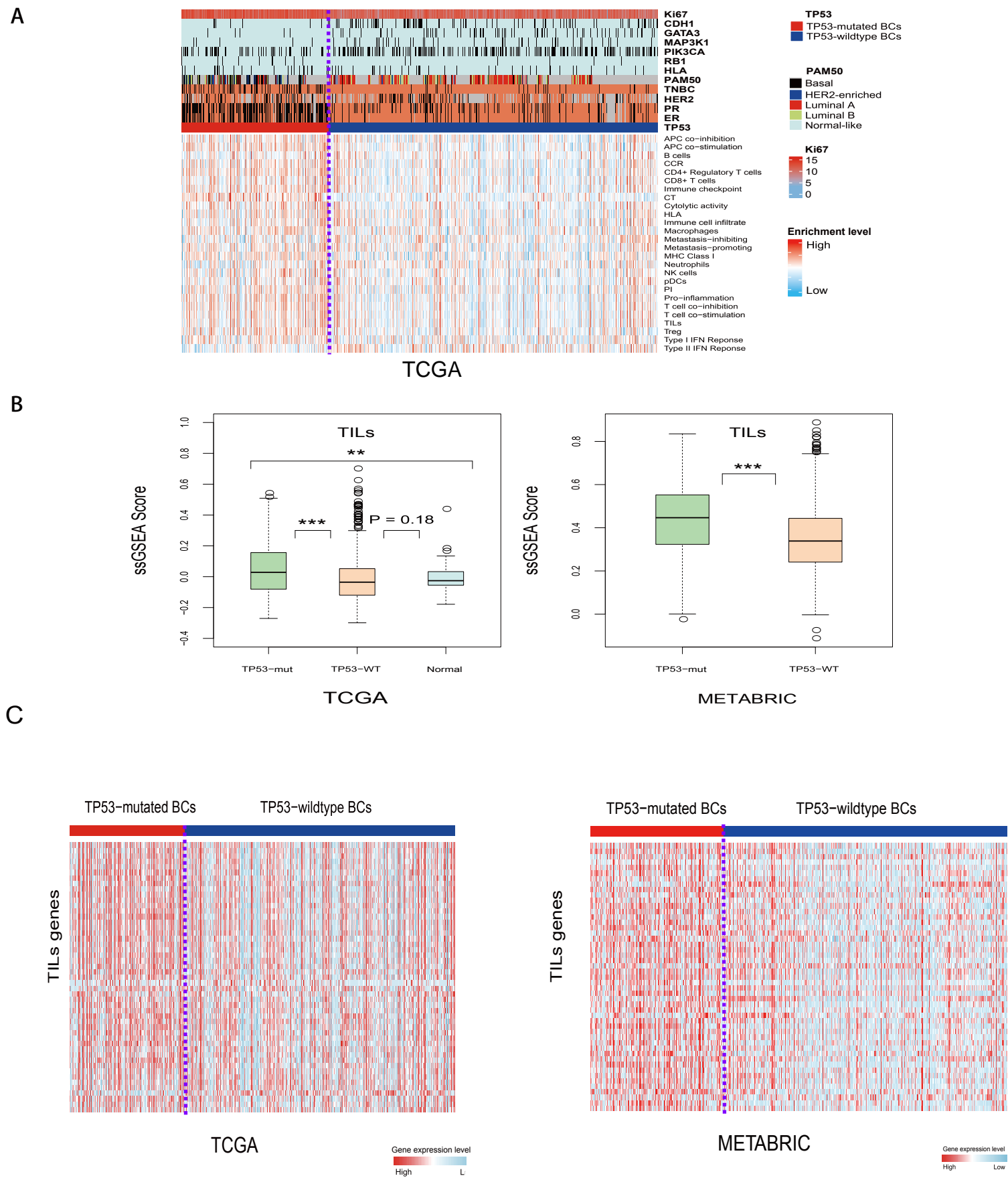

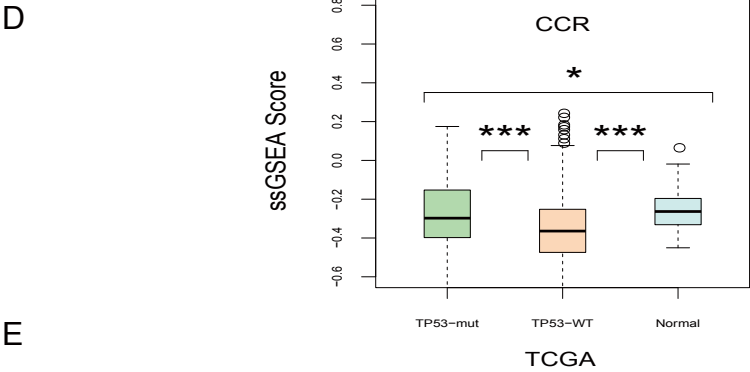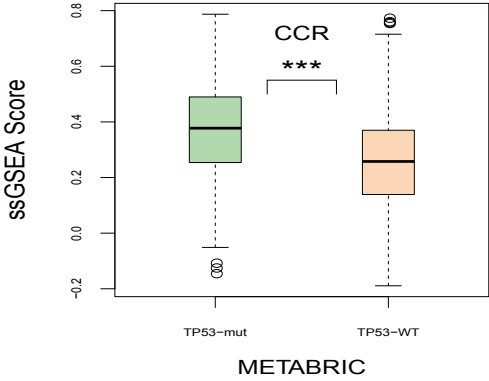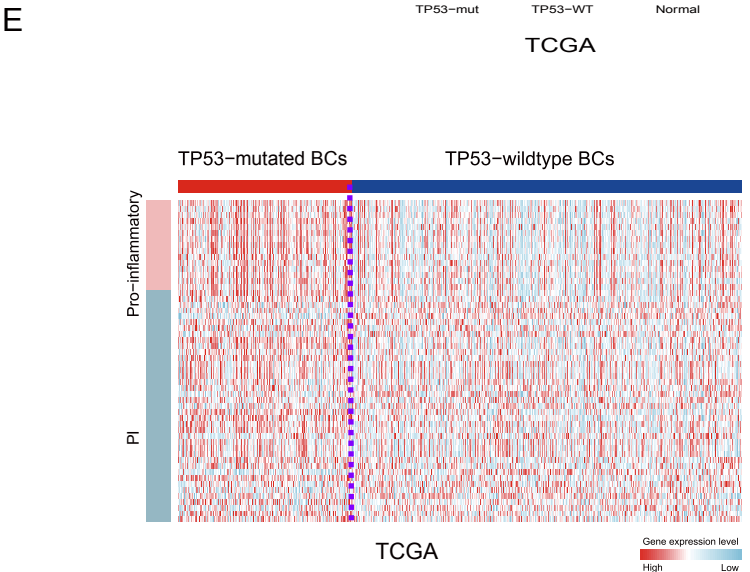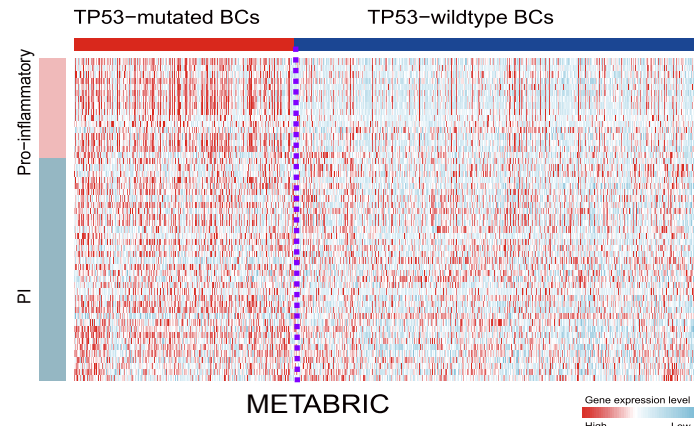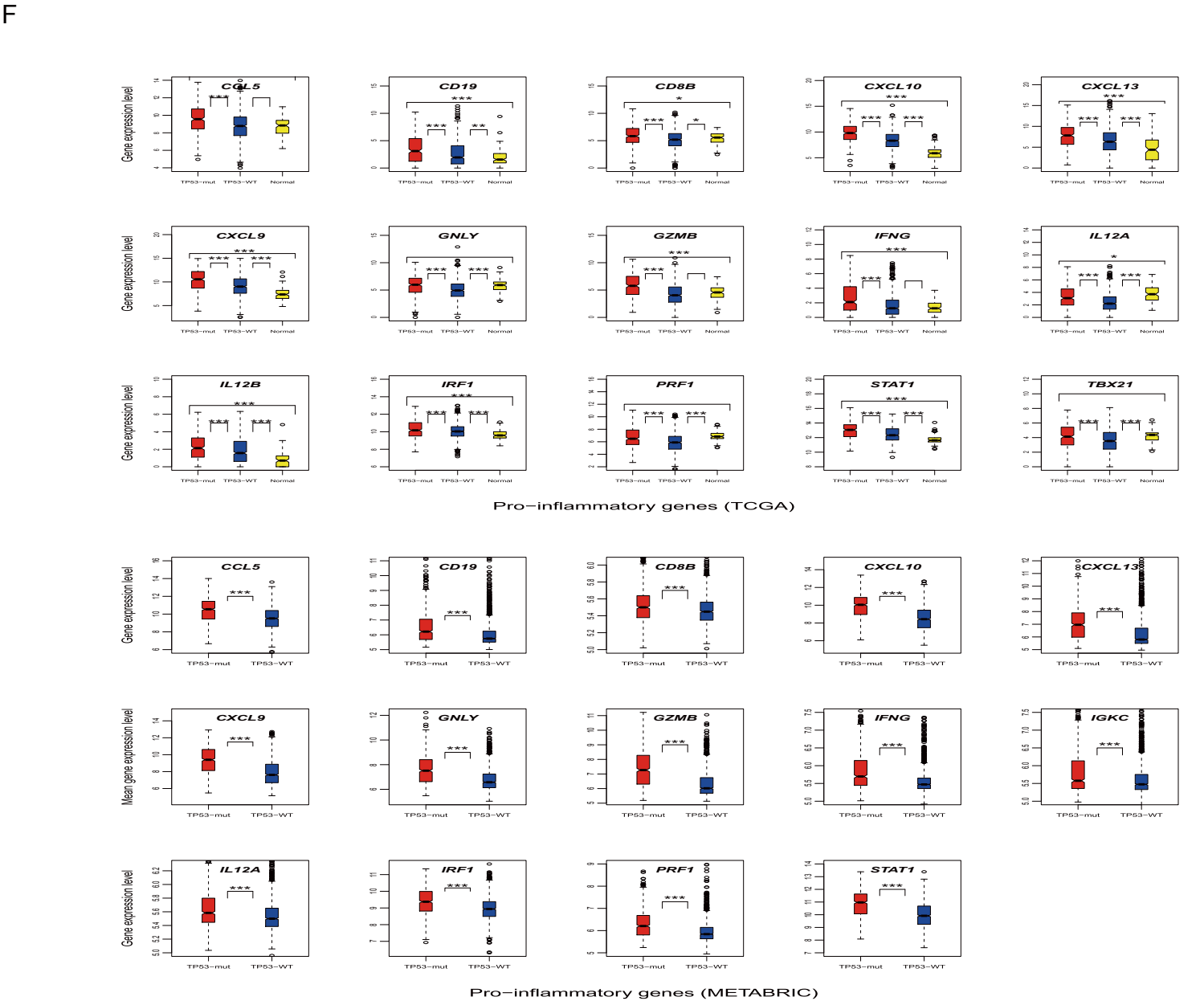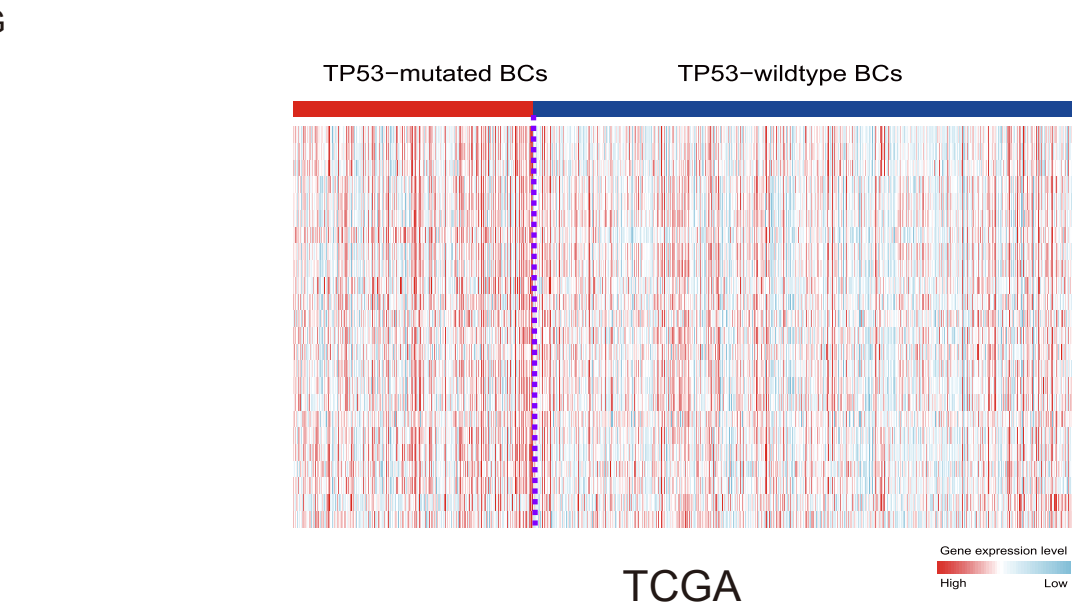

Supplement: Supplementary Materials — Supplementary Tables. Table S1: the list of 26 immune signatures and related gene sets. Table S2: sample sizes of breast cancers. Table S3: ssGSEA scores of immune signature in TCGA and METABRIC. Table S4: primer sequences used for real time quantity PCR. Table S5: comparison of the enrichment levels of 15 immune cell types and function signatures between two classes of samples. Table S6: comparison of the enrichment levels of the tumor-infiltrating lymphocytes signature between two classes of samples. Table S7: comparison of the enrichment levels of the cytokine and cytokine receptor signature between two classes of samples. Table S8: comparison of the enrichment levels of the inflammation-promoting and parainflammation (PI) signatures between two classes of samples. Table S9: comparison of the enrichment levels of the HLA signature between two classes of samples. Table S10: comparisons of the ssGSEA scores of immune signatures between TP53-mutated and TP53-wildtype BCs and their associations with survival prognosis in BC. Table S11: comparisons of the expression levels of immune genes between TP53-mutated and TP53-wildtype BCs and their associations with survival prognosis in BC. Table S12: comparisons of the expression levels of genes and their protein products between TP53-mutated and TP53-wildtyped BCs. Table S13: comparisons of the enrichment levels of immune signatures between TP53-mutated and TP53-wildtype BCs within the ER+ subtype of BC. Table S14: comparisons of the enrichment levels of immune signatures between TP53-mutated and TP53-wildtype BCs within the HER2- subtype of BC. Table S15: comparisons of the enrichment levels of immune signatures between TP53-mutated and TP53-wildtype BCs within the 100% tumor purity of BC. Table S16: comparisons of the enrichment levels of the cancer-testis signature between two classes of samples. Table S17: comparisons of the enrichment levels of the Treg signature between two classes of samples. Table S18: comparisons [file 5952836.f1.zip › 5952836.f1/Supplementary Figure S1.pdf]

Figure S2

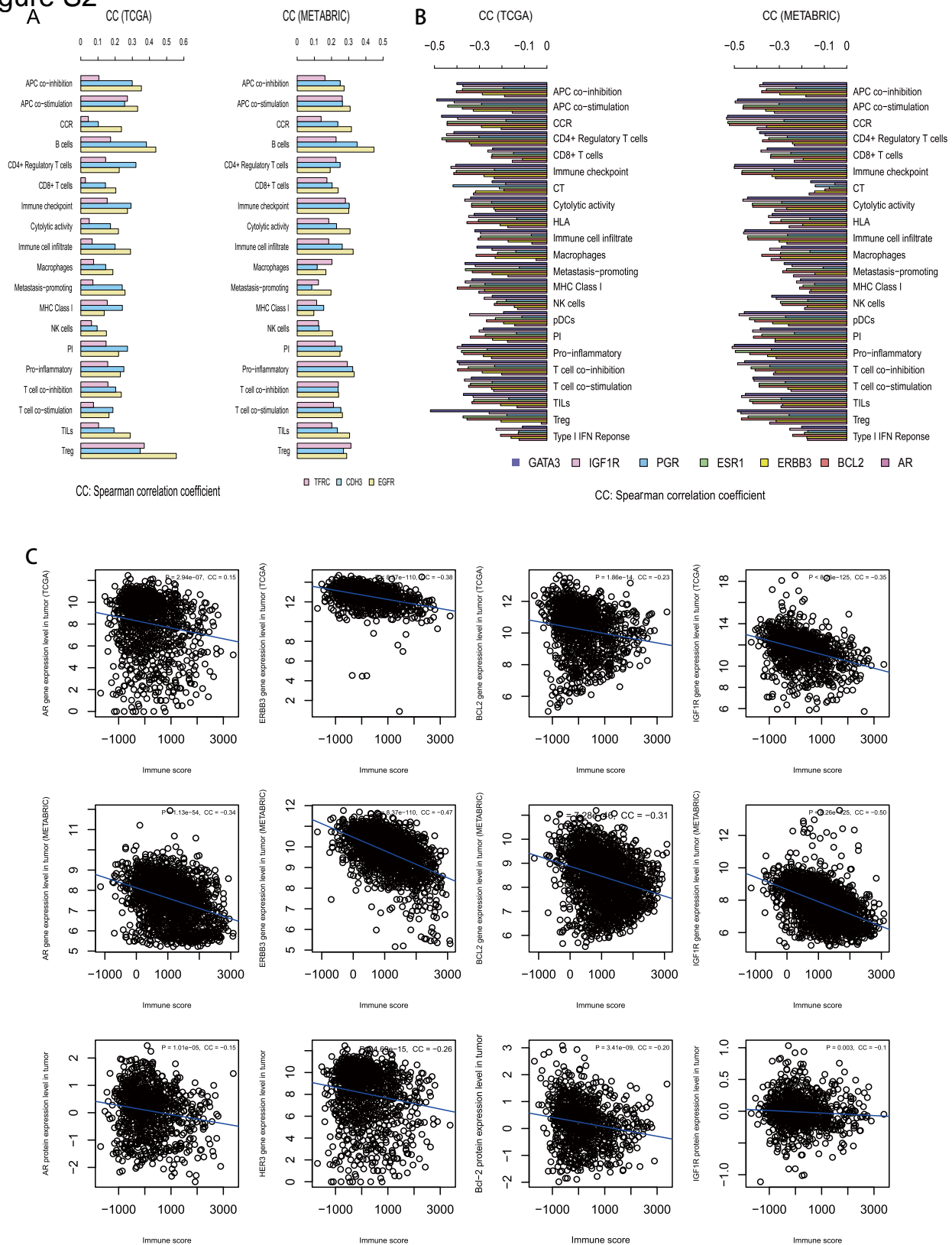

Supplement: Supplementary Materials — Supplementary Tables. Table S1: the list of 26 immune signatures and related gene sets. Table S2: sample sizes of breast cancers. Table S3: ssGSEA scores of immune signature in TCGA and METABRIC. Table S4: primer sequences used for real time quantity PCR. Table S5: comparison of the enrichment levels of 15 immune cell types and function signatures between two classes of samples. Table S6: comparison of the enrichment levels of the tumor-infiltrating lymphocytes signature between two classes of samples. Table S7: comparison of the enrichment levels of the cytokine and cytokine receptor signature between two classes of samples. Table S8: comparison of the enrichment levels of the inflammation-promoting and parainflammation (PI) signatures between two classes of samples. Table S9: comparison of the enrichment levels of the HLA signature between two classes of samples. Table S10: comparisons of the ssGSEA scores of immune signatures between TP53-mutated and TP53-wildtype BCs and their associations with survival prognosis in BC. Table S11: comparisons of the expression levels of immune genes between TP53-mutated and TP53-wildtype BCs and their associations with survival prognosis in BC. Table S12: comparisons of the expression levels of genes and their protein products between TP53-mutated and TP53-wildtyped BCs. Table S13: comparisons of the enrichment levels of immune signatures between TP53-mutated and TP53-wildtype BCs within the ER+ subtype of BC. Table S14: comparisons of the enrichment levels of immune signatures between TP53-mutated and TP53-wildtype BCs within the HER2- subtype of BC. Table S15: comparisons of the enrichment levels of immune signatures between TP53-mutated and TP53-wildtype BCs within the 100% tumor purity of BC. Table S16: comparisons of the enrichment levels of the cancer-testis signature between two classes of samples. Table S17: comparisons of the enrichment levels of the Treg signature between two classes of samples. Table S18: comparisons [file 5952836.f1.zip › 5952836.f1/Supplementary Figure S2.pdf]

A

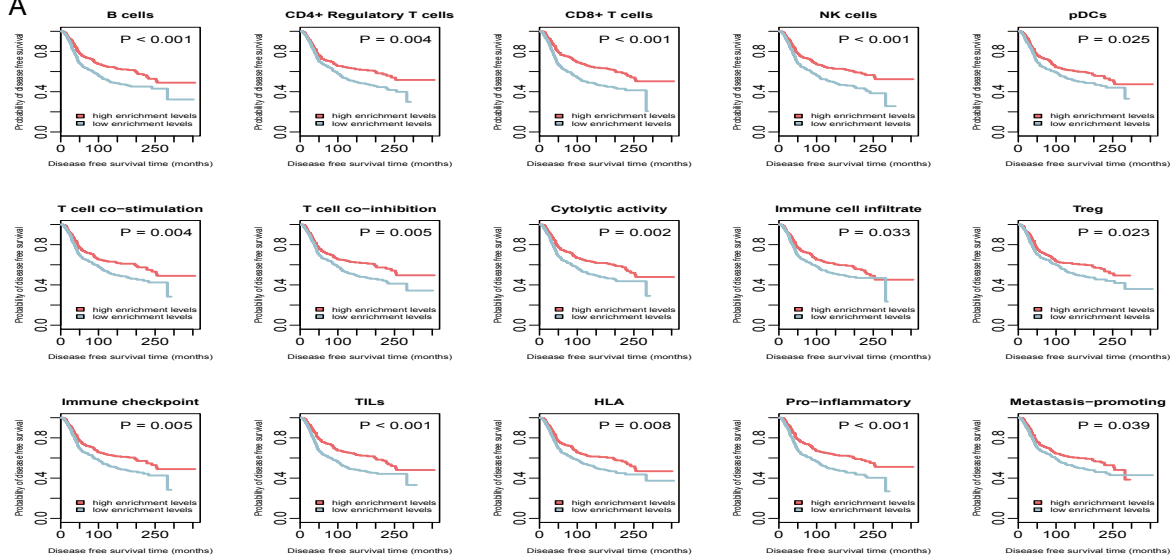

B

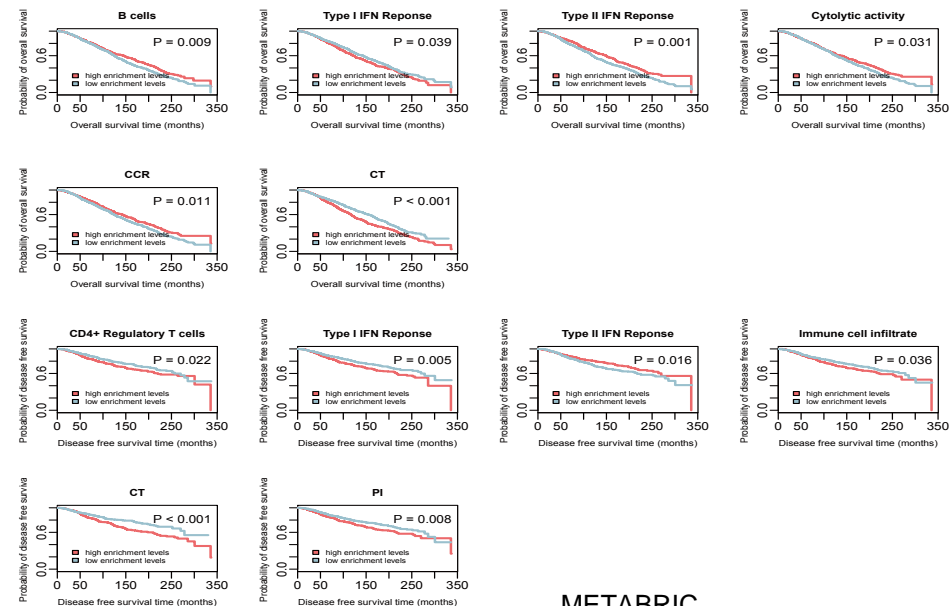

METABRIC

METABRIC

C

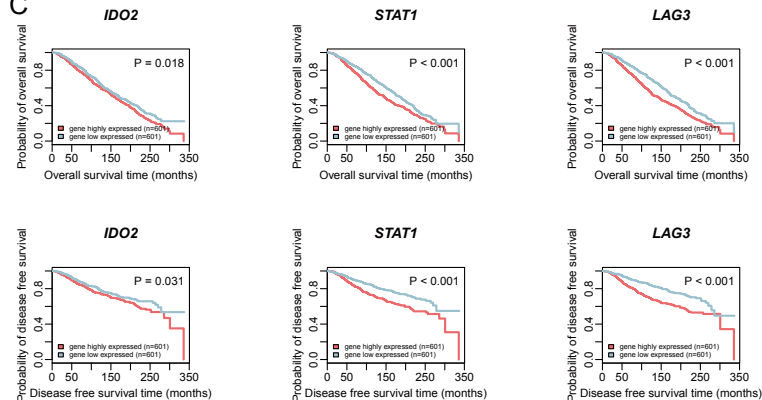

METABRIC

Supplement: Supplementary Materials — Supplementary Tables. Table S1: the list of 26 immune signatures and related gene sets. Table S2: sample sizes of breast cancers. Table S3: ssGSEA scores of immune signature in TCGA and METABRIC. Table S4: primer sequences used for real time quantity PCR. Table S5: comparison of the enrichment levels of 15 immune cell types and function signatures between two classes of samples. Table S6: comparison of the enrichment levels of the tumor-infiltrating lymphocytes signature between two classes of samples. Table S7: comparison of the enrichment levels of the cytokine and cytokine receptor signature between two classes of samples. Table S8: comparison of the enrichment levels of the inflammation-promoting and parainflammation (PI) signatures between two classes of samples. Table S9: comparison of the enrichment levels of the HLA signature between two classes of samples. Table S10: comparisons of the ssGSEA scores of immune signatures between TP53-mutated and TP53-wildtype BCs and their associations with survival prognosis in BC. Table S11: comparisons of the expression levels of immune genes between TP53-mutated and TP53-wildtype BCs and their associations with survival prognosis in BC. Table S12: comparisons of the expression levels of genes and their protein products between TP53-mutated and TP53-wildtyped BCs. Table S13: comparisons of the enrichment levels of immune signatures between TP53-mutated and TP53-wildtype BCs within the ER+ subtype of BC. Table S14: comparisons of the enrichment levels of immune signatures between TP53-mutated and TP53-wildtype BCs within the HER2- subtype of BC. Table S15: comparisons of the enrichment levels of immune signatures between TP53-mutated and TP53-wildtype BCs within the 100% tumor purity of BC. Table S16: comparisons of the enrichment levels of the cancer-testis signature between two classes of samples. Table S17: comparisons of the enrichment levels of the Treg signature between two classes of samples. Table S18: comparisons [file 5952836.f1.zip › 5952836.f1/Supplementary Figure S3.pdf]

Figure 4  
A

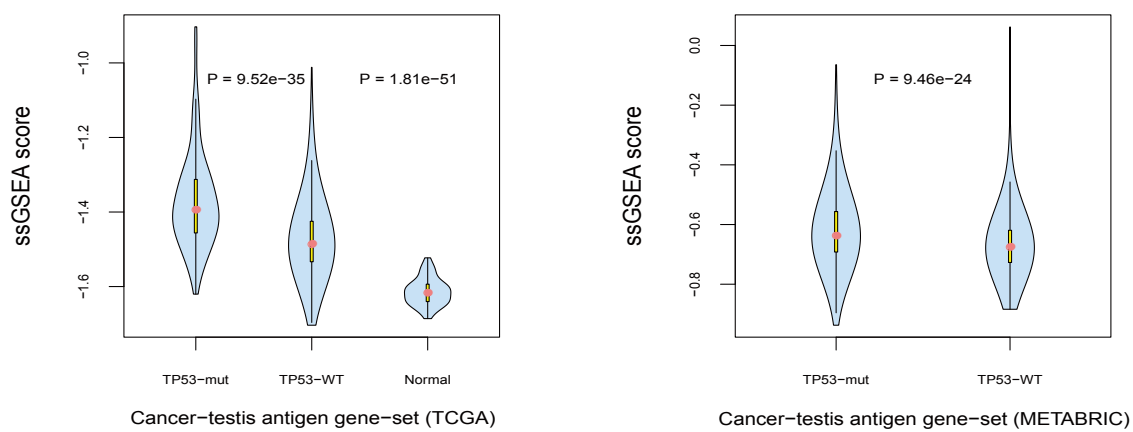

B

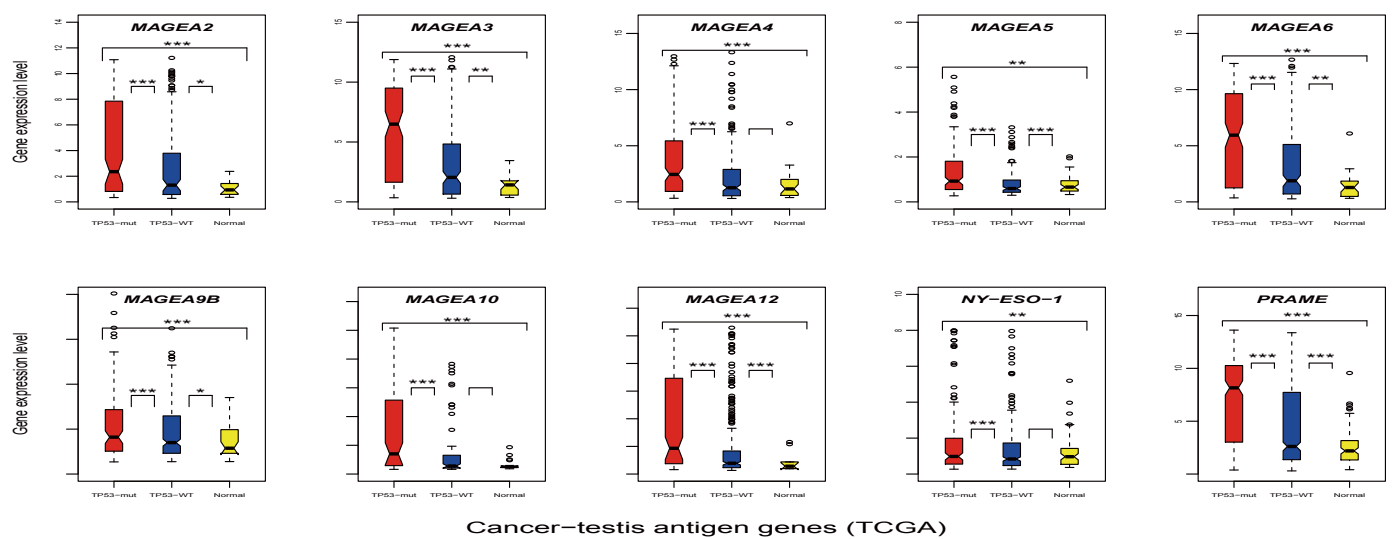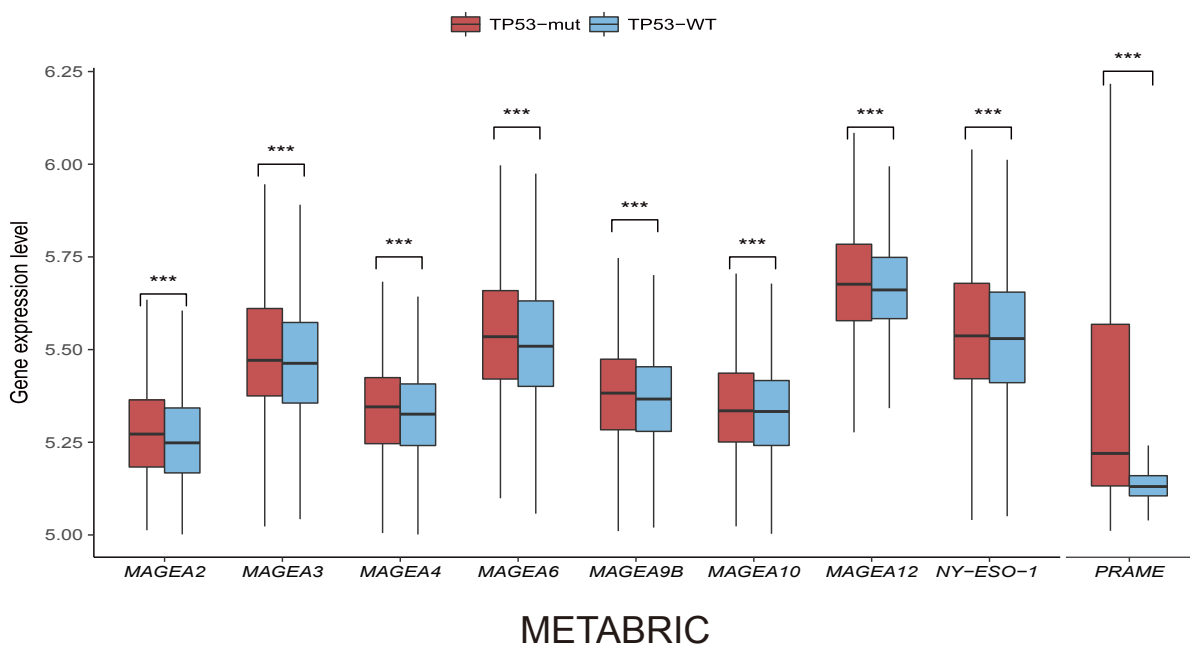

C

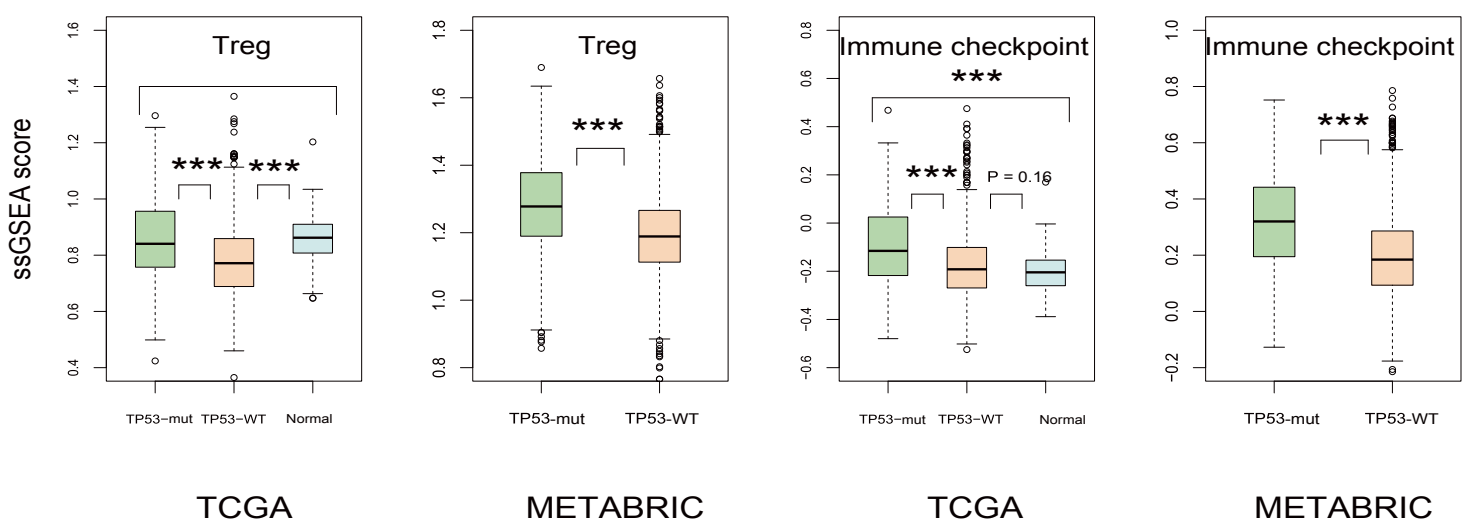

D

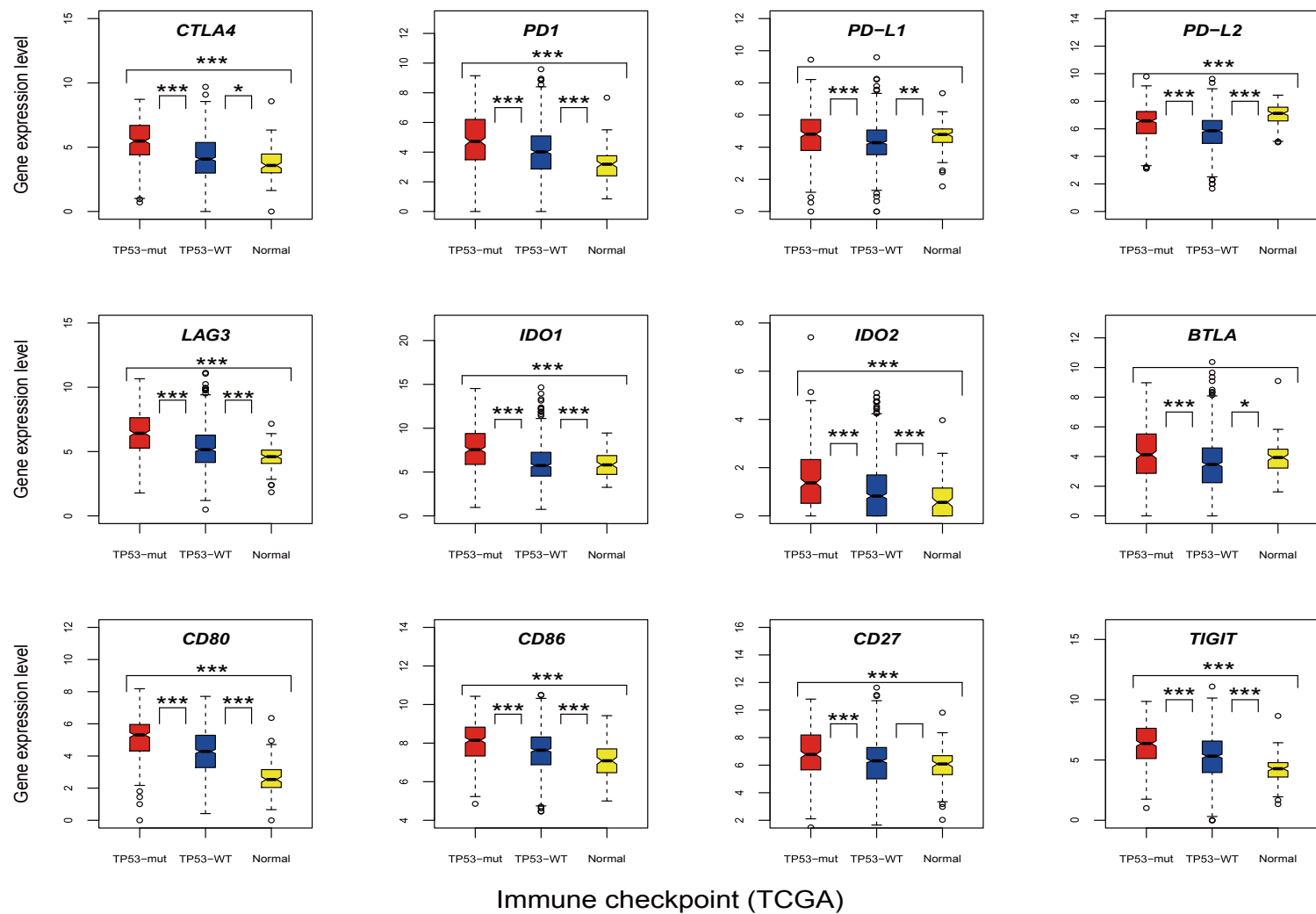

E

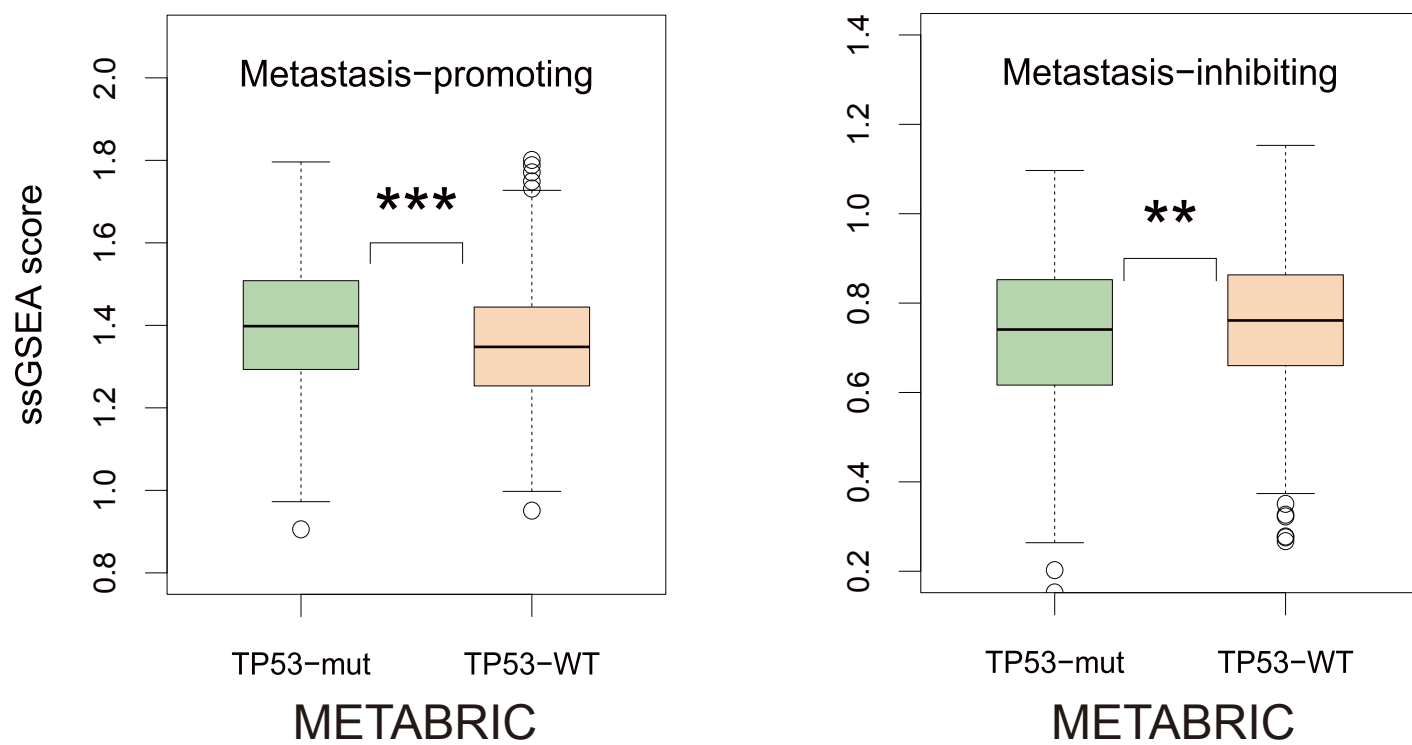

Supplement: Supplementary Materials — Supplementary Tables. Table S1: the list of 26 immune signatures and related gene sets. Table S2: sample sizes of breast cancers. Table S3: ssGSEA scores of immune signature in TCGA and METABRIC. Table S4: primer sequences used for real time quantity PCR. Table S5: comparison of the enrichment levels of 15 immune cell types and function signatures between two classes of samples. Table S6: comparison of the enrichment levels of the tumor-infiltrating lymphocytes signature between two classes of samples. Table S7: comparison of the enrichment levels of the cytokine and cytokine receptor signature between two classes of samples. Table S8: comparison of the enrichment levels of the inflammation-promoting and parainflammation (PI) signatures between two classes of samples. Table S9: comparison of the enrichment levels of the HLA signature between two classes of samples. Table S10: comparisons of the ssGSEA scores of immune signatures between TP53-mutated and TP53-wildtype BCs and their associations with survival prognosis in BC. Table S11: comparisons of the expression levels of immune genes between TP53-mutated and TP53-wildtype BCs and their associations with survival prognosis in BC. Table S12: comparisons of the expression levels of genes and their protein products between TP53-mutated and TP53-wildtyped BCs. Table S13: comparisons of the enrichment levels of immune signatures between TP53-mutated and TP53-wildtype BCs within the ER+ subtype of BC. Table S14: comparisons of the enrichment levels of immune signatures between TP53-mutated and TP53-wildtype BCs within the HER2- subtype of BC. Table S15: comparisons of the enrichment levels of immune signatures between TP53-mutated and TP53-wildtype BCs within the 100% tumor purity of BC. Table S16: comparisons of the enrichment levels of the cancer-testis signature between two classes of samples. Table S17: comparisons of the enrichment levels of the Treg signature between two classes of samples. Table S18: comparisons [file 5952836.f1.zip › 5952836.f1/Supplementary Figure S4.pdf]

Figure S5

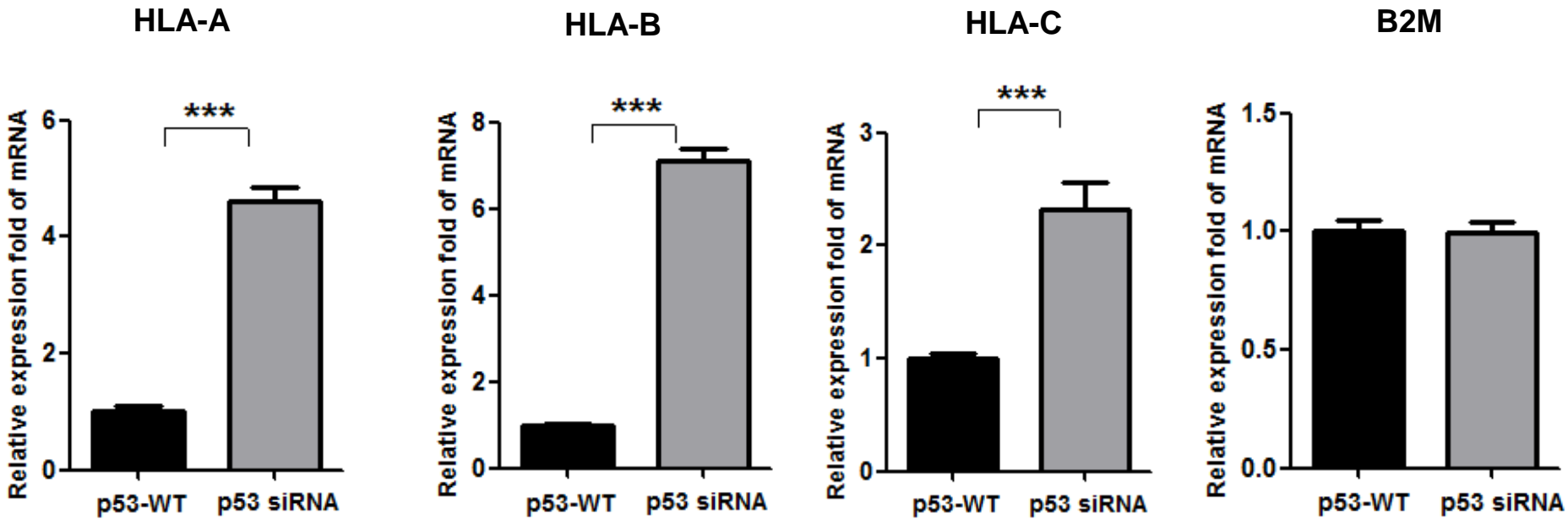

Supplement: Supplementary Materials — Supplementary Tables. Table S1: the list of 26 immune signatures and related gene sets. Table S2: sample sizes of breast cancers. Table S3: ssGSEA scores of immune signature in TCGA and METABRIC. Table S4: primer sequences used for real time quantity PCR. Table S5: comparison of the enrichment levels of 15 immune cell types and function signatures between two classes of samples. Table S6: comparison of the enrichment levels of the tumor-infiltrating lymphocytes signature between two classes of samples. Table S7: comparison of the enrichment levels of the cytokine and cytokine receptor signature between two classes of samples. Table S8: comparison of the enrichment levels of the inflammation-promoting and parainflammation (PI) signatures between two classes of samples. Table S9: comparison of the enrichment levels of the HLA signature between two classes of samples. Table S10: comparisons of the ssGSEA scores of immune signatures between TP53-mutated and TP53-wildtype BCs and their associations with survival prognosis in BC. Table S11: comparisons of the expression levels of immune genes between TP53-mutated and TP53-wildtype BCs and their associations with survival prognosis in BC. Table S12: comparisons of the expression levels of genes and their protein products between TP53-mutated and TP53-wildtyped BCs. Table S13: comparisons of the enrichment levels of immune signatures between TP53-mutated and TP53-wildtype BCs within the ER+ subtype of BC. Table S14: comparisons of the enrichment levels of immune signatures between TP53-mutated and TP53-wildtype BCs within the HER2- subtype of BC. Table S15: comparisons of the enrichment levels of immune signatures between TP53-mutated and TP53-wildtype BCs within the 100% tumor purity of BC. Table S16: comparisons of the enrichment levels of the cancer-testis signature between two classes of samples. Table S17: comparisons of the enrichment levels of the Treg signature between two classes of samples. Table S18: comparisons [file 5952836.f1.zip › 5952836.f1/Supplementary Figure S5.pdf]
